# Construction of EGFR-Targeted Triptolide Liposomes Using Uniform Design Optimization and Therapeutic Evaluation in Gliomas

**DOI:** 10.3390/ph19050731

**Published:** 2026-05-06

**Authors:** Huiqing Que, Wei Li, Ziting Li, Lihong Ma, Junyong Han, Shenmin Liu, Xiaomei Xu, Liping Qian, Wenjin Lin, Hongxu Liu

**Affiliations:** 1 Fujian Key Laboratory of Medical Analysis, Fujian Academy of Medical Sciences, Fuzhou 350001, Chinalihongma@fjms.ac.cn (L.M.); hanjunyong@fjms.ac.cn (J.H.);; 2 College of Pharmacy, Fujian University of Traditional Chinese Medicine, Fuzhou 350122, China; 2230456058@fjtcm.edu.cn; 3 College of Bee Science and Biomedicine, Fujian Agriculture and Forestry University, Fuzhou 350002, China; 000q825014@fafu.edu.cn

**Keywords:** triptolide, liposomes, EGFR targeting, glioma therapy, uniform design optimization, therapeutic evaluation

## Abstract

**Background/Objectives:** Triptolide (TP), a potent natural diterpenoid, exhibits anti-glioma activity, but faces significant clinical translation challenges, including poor water solubility, systemic toxicity such as hepatotoxicity, and inadequate tumor targeting. This study aimed to develop a novel epidermal growth factor receptor (EGFR)-targeted liposomal formula-tion, designated as TP-CTX-Lip (where CTX denotes cetuximab), to enhance the deliv-ery efficiency and therapeutic window of TP. **Methods:** The formulation was optimized using a uniform design approach (four factors, six levels) and prepared via thin-film hydra-tion–ultrasonication. The encapsulation of TP was supported by Fourier transform in-frared spectroscopy (FTIR) and thermal analysis (DSC/TGA), which revealed molecu-lar interactions (e.g., hydrogen bonding) with lipid components and a marked en-hancement in thermal stability, consistent with successful incorporation into the lipo-somal bilayer. The physicochemical properties, including the size, polydispersity index (PDI), zeta potential, encapsulation efficiency, and drug loading, were characterized. In vitro release kinetics were evaluated in phosphate buffer (pH 7.4), and cytotoxicity was assessed in high-EGFR (U87-MG) and low-EGFR (SW1088) glioma cells. In vivo efficacy and developmental toxicity were investigated using zebrafish models. The op-timized TP-CTX-Lip demonstrated favorable characteristics: size = 131.3 ± 4.5 nm, PDI = 0.24 ± 0.006, zeta potential = −23.37 ± 0.27 mV, encapsulation efficiency = 85.83% ± 1.81%, and drug loading = 13%. In vitro release followed first-order kinetics dominated by Higuchi diffusion (79.0% ± 4% at 24 h). After 48 h of treatment, TP-CTX-Lip exhib-ited significantly enhanced cytotoxicity in U87-MG cells (IC50 = 10.4 ± 0.2 nM), com-pared with IC50 values of 42.8 nM in SW1088 cells and 45.3 nM for non-targeted lipo-somes. In the 3T3-L1 non-cancerous cell line, the 48 h IC50 value of TP-CTX-Lip (8.433 ± 0.954µM) was higher than that of the TP solution (2.173 ± 0.181µM) but lower than that of TP-Lip (25.78 ± 2.691µM). Specifically, in 3T3-L1 cells, the 48 h IC50 of TP-CTX-Lip (8.43 µM) was approximately 4-fold higher than that of free TP (2.17 µM), confirming its substantially reduced cytotoxicity against non-cancerous cells. **Results:** In comparison to TP-Lip and free FITC solution, the uptake rate of TP-CTX-Lip in U87-MG cells exhibited a significantly higher level. Specifically, the uptake rate for the TP-CTX-Lip group (57.46 ± 5.44%) was statistically significantly higher than that of TP-Lip (13.7 ± 2.33%) and the free FITC solution group (20.97 ± 1.60%) (*p* < 0.01). In zebrafish, TP-CTX-Lip reduced developmental toxicity, with LC50 increased 1.26 times to 5.733 μg/mL, and suppressed orthotopic U87-MG xenograft growth (*p* < 0.001), in-dicating an improved therapeutic window as reflected by the LC50/IC50 ratio. **Conclusions:** the EGFR-targeted TP-CTX-Lip significantly enhances the tumor selectivity and safety of TP, providing a promising strategy for targeted glioma therapy.

## 1. Introduction

Gliomas are among the most aggressive and lethal types of brain tumors. Their incidence rate is approximately 5.26 per 100,000 individuals annually [1,2,3]. The World Health Organization (WHO) classifies gliomas into various grades. Among these, glioblastoma multiforme (GBM) is the most common and malignant form. GBM is characterized by a poor prognosis, with a median survival of only 15 months post diagnosis [4,5,6]. Current treatment modalities include surgical resection, radiation therapy, and chemotherapy. However, these often yield limited efficacy. This limitation stems from the tumor’s infiltrative nature and the blood–brain barrier (BBB), which restricts drug delivery [7,8,9]. Moreover, conventional chemotherapeutic agents frequently exhibit systemic toxicity, leading to adverse effects such as hepatotoxicity and compromised patient quality of life [10,11]. These challenges underscore the urgent need for innovative therapeutic strategies. Such strategies should enhance drug delivery specifically to glioma cells while minimizing systemic side effects. Triptolide (TP), a natural diterpenoid derived from the plant Tripterygium wilfordii, has demonstrated potent anti-glioma activity [12,13,14]; however, its clinical application is hindered by poor water solubility and significant systemic toxicity [15,16,17]. Therefore, developing targeted delivery systems for TP could potentially improve its therapeutic efficacy and safety profile in glioma treatment [18,19,20].

TP is known for its potent anti-cancer properties, particularly against gliomas [21]. The molecular mechanisms underlying TP’s anti-glioma activity involve multiple processes. These include the induction of apoptosis and the inhibition of cell proliferation. TP also modulates various signaling pathways, such as the NF-κB and MAPK pathways. These pathways play crucial roles in cell survival and proliferation. Therefore, TP is a promising candidate for glioma treatment [22,23,24]. However, the clinical application of TP is hindered by its poor water solubility, systemic toxicity—particularly hepatotoxicity—and lack of tumor specificity, which limits its therapeutic efficacy [25,26].

Recent studies have highlighted the potential of targeted drug delivery systems to overcome these limitations. For instance, the use of EGFR targeting strategies has gained attention due to the overexpression of EGFR in many glioma cells [19,27,28]. By conjugating TP to EGFR-targeted carriers, researchers aim to enhance its selective delivery to tumor cells while minimizing off-target effects. Despite these advancements, challenges remain in optimizing the delivery systems for improved bioavailability and reduced toxicity [13,17,29]. Current research is focused on developing novel formulations, such as immunoliposomes, that can effectively encapsulate TP and facilitate its targeted delivery to glioma cells, thereby improving its therapeutic window and overall efficacy in clinical settings.

This study aims to address the significant challenges associated with the clinical translation of TP, a natural diterpenoid known for its anti-glioma properties. The primary objective is to develop a novel EGFR-targeted immunoliposome, termed TP-CTX-Lip, designed to enhance the delivery efficiency of TP while minimizing systemic toxicity and improving tumor targeting. By employing a systematic approach that includes uniform design methodology for optimization and thin-film hydration–ultrasonication for preparation, this research seeks to create a formulation that not only encapsulates TP effectively but also exhibits favorable physicochemical properties. The significance of this study lies in its potential to improve therapeutic outcomes for glioma patients by increasing the selectivity and safety of TP, thereby overcoming the limitations of its poor water solubility and associated hepatotoxicity. Ultimately, the findings of this study may provide new strategies for more effective targeted therapy for gliomas, promoting advancements in glioma treatment methods.

## 2. Results

### 2.1. Construction and Characterization of TP-CTX-Lip

#### 2.1.1. Preparation of TP-Lip

**Figure 1:**
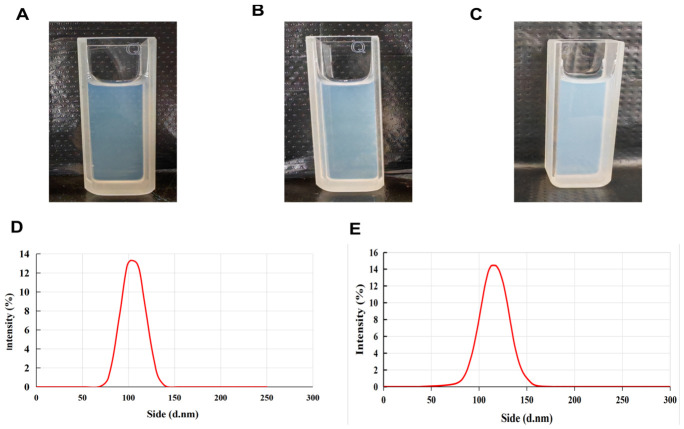

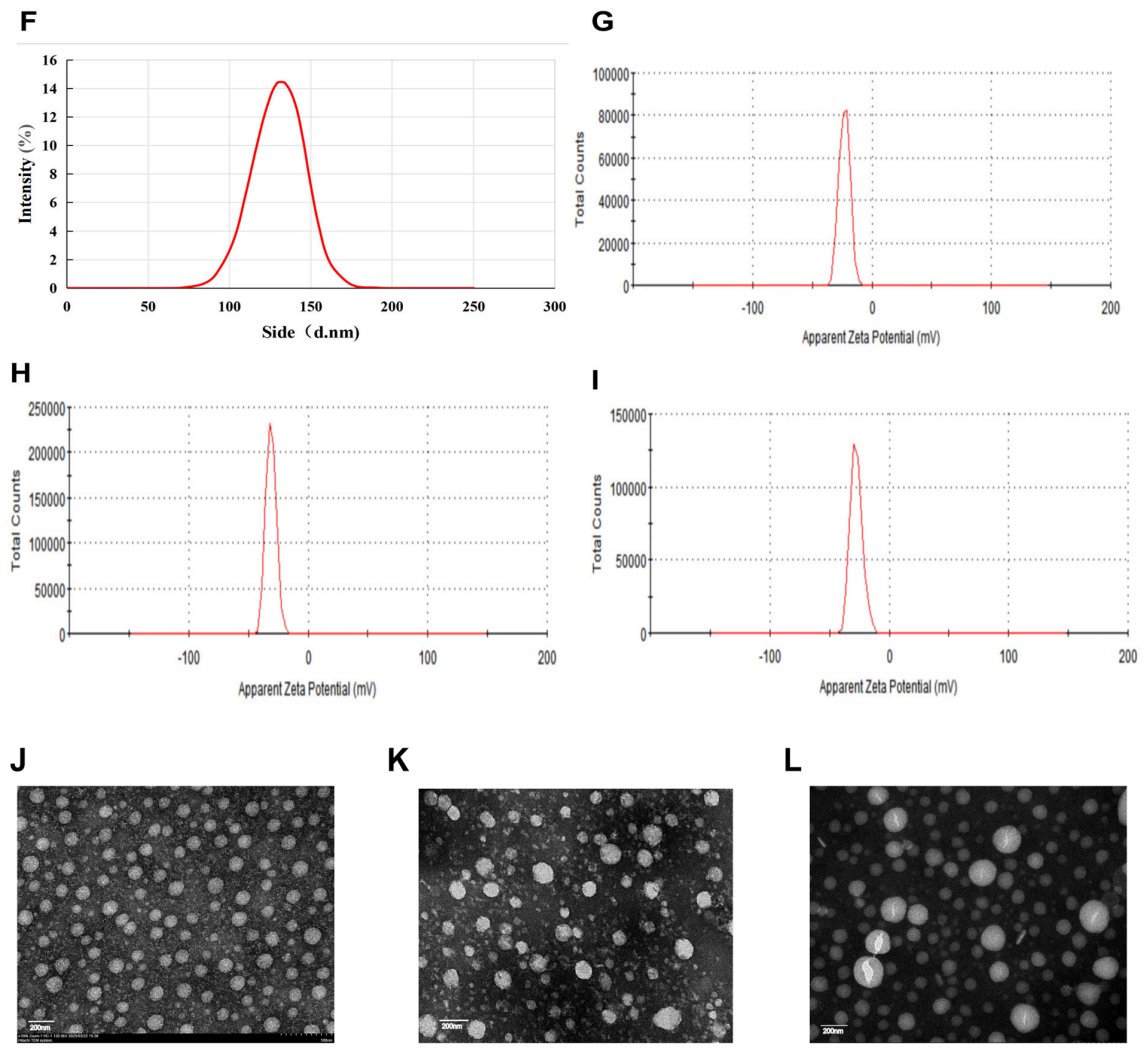
Analysis of the physical and chemical properties of liposome formulations, with sections (**A**–**C**) displaying the appearance of Blank-Lip, TP-Lip, and TP-CTX-Lip at 25 °C. Sections (**D**–**F**) show the particle size distribution curves of Blank-Lip, TP-Lip, and TP-CTX-Lip. Sections (**G**–**I**) display the ζ potential measurements of Blank-Lip, TP-Lip, and TP-CTX-Lip. Sections (**J**–**L**) present the TEM images of Blank-Lip, TP-Lip, and TP-CTX-Lip, with a scale bar of 100 nm. (*n* = 3).

A uniform lipid film was generated by means of rotary evaporation and hydrated with PBS. This was followed by ultrasonic emulsification to prepare a semi-transparent suspension of liposomes with characteristic pale blue opalescence (Figure 1A–C). This method aimed to address the inherent poor water solubility and to potentially enhance its drug-like properties for further evaluation.

A four-factor uniform design (UD) system was adopted to conduct six experiments (Table 1). Quantitative analysis was carried out on the key formulation influencing factors: the dosage of Hydrogenated Soybean Phospholipids {HSPC (X_1_)}, the ratio of HSPC to cholesterol (X_2_), the ratio of HSPC to DSPE-PEG_2000_-Mal (X_3_), and the ratio of HSPC to TP (X_4_), based on the experimental data (Table 2). The dosage of HSPC showed a significant positive impact (β = 0.986, *p* = 0.013), with an increase of 0.01 millimoles leading to an approximately 1192-point rise in the evaluation score. The molar ratio of HSPC to cholesterol demonstrated a synergistic enhancing effect (β = 3.974, *p* = 0.036), which is conducive to optimizing the membrane arrangement [30]. Additionally, the content of DSPE-PEG_2000_-Mal had a negative influence on the score (β = −2.432, *p* = 0.035), mainly attributed to the steric hindrance effect of PEG [31]. However, the loading ratio of TP showed no statistically significant difference (*p* = 0.819), indicating that within the studied range, the dosage of TP was not a key determinant of the outcome under these experimental conditions. Based on the above results, the following predictive regression model has been established (R^2^ = 0.998):

(1) $$\begin{array}{c} Y\;=\;1192.347X_{1}\;+\;3.974X_{2}\;-\;2.432X_{3}\;+\;0.024X_{4}\;+\;190.697 \end{array}$$

The optimal parameters derived from the regression model are as follows: HSPC amount, 0.07 mmol; ratios of components—HSPC to cholesterol, 6:1; HSPC to DSPE-PEG_2000_-Mal, 1:1; and HSPC to TP, 4:1. The TP-Lip prepared according to this optimized formula have the characteristics listed in Table 3.

#### 2.1.2. Incubation of TP-CTX-Lip

After thiolation, CTX was bound with TP-Lip to form TP-CTX-Lip. The optimal conditions for the CTX thiolation reaction were as follows: CTX and 2-IT were thoroughly mixed at a molar ratio of 1:3 at room temperature and incubated for 3 h. A cysteine standard curve (Y = 0.0642x + 10^−5^; R^2^ = 0.9995) was established. Using this curve, the concentration of free sulfhydryl groups (-SH) in CTX-SH was measured as 1.06 millimoles per liter, with a thiolation modification efficiency of 87.65%. After thiolation, CTX-SH underwent a Michael addition reaction with DSPE-PEG_2000_-Mal contained in TP-Lip at a molar ratio of 10:1 to form stable thioether bonds, resulting in the formation of immunoliposomes (TP-CTX-Lip).

#### 2.1.3. Characterization of TP-CTX-Lip

Three types of liposomes—Blank-Lip, TP-Lip, and TP-CTX-Lip—were prepared using an optimized formulation. The particle sizes of these liposomes were analyzed using dynamic light scattering (DLS). The results showed that all three had narrow hydrodynamic particle sizes, and the particle size distribution exhibited a normal distribution characteristic (Figure 1D–F). Additionally, the polydispersity index (PDI) of all formulations was lower than 0.25 (Table 4), indicating that the liposome formulations were highly homogeneous. At the same time, the zeta potential test results showed that all liposomes carried strong negative charges (Figure 1G–I). Due to this high negative charge, electrostatic repulsion was enhanced. This effectively inhibited particle aggregation. It also improved the colloidal dispersion stability of the liposomes in solution [32].

The morphological characteristics of the liposomes were evaluated using transmission electron microscopy (TEM). Figure 1J–L show images at the nanoscale (approximately 100 nanometers). The particles of all three formulations are spherical in shape. The particle size of Blank-Lip is the smallest, that of TP-Lip is slightly larger, and TP-CTX-Lip has the largest particle size. TEM observations also revealed that some TP-CTX-Lip particles exhibited slightly irregular shapes and rough surfaces (Figure 1L). The increase in particle size and subtle changes in morphology correspond to the successful binding of CTX to the surface of pre-formed TP liposomes, which may lead to the formation of an additional surface layer or changes in the topological structure. Structurally, TP may interact with the phospholipid/cholesterol bilayer via non-covalent forces. These potential interactions include hydrogen bonding and van der Waals forces. However, the possibility of covalent bond formation cannot be ruled out. The trend of gradually increasing particle size from Blank-Lip to TP-Lip to TP-CTX-Lip was reflected in both DLS data and TEM images, indicating that TP-CTX-Lip was successfully prepared and its physical and chemical properties remained intact.

In summary, after chemical modification through sulfhydryl groups and subsequent directed coupling, CTX was effectively fixed on the surface of liposomes. This strategy resulted in the construction of a functionalized immunoliposome system suitable for targeted delivery [33,34]. Importantly, this system exhibited consistent and controllable morphological characteristics and particle size distribution, providing a solid experimental foundation for subsequent evaluation of its targeted delivery efficiency and anti-tumor activity.

**Figure 2:**
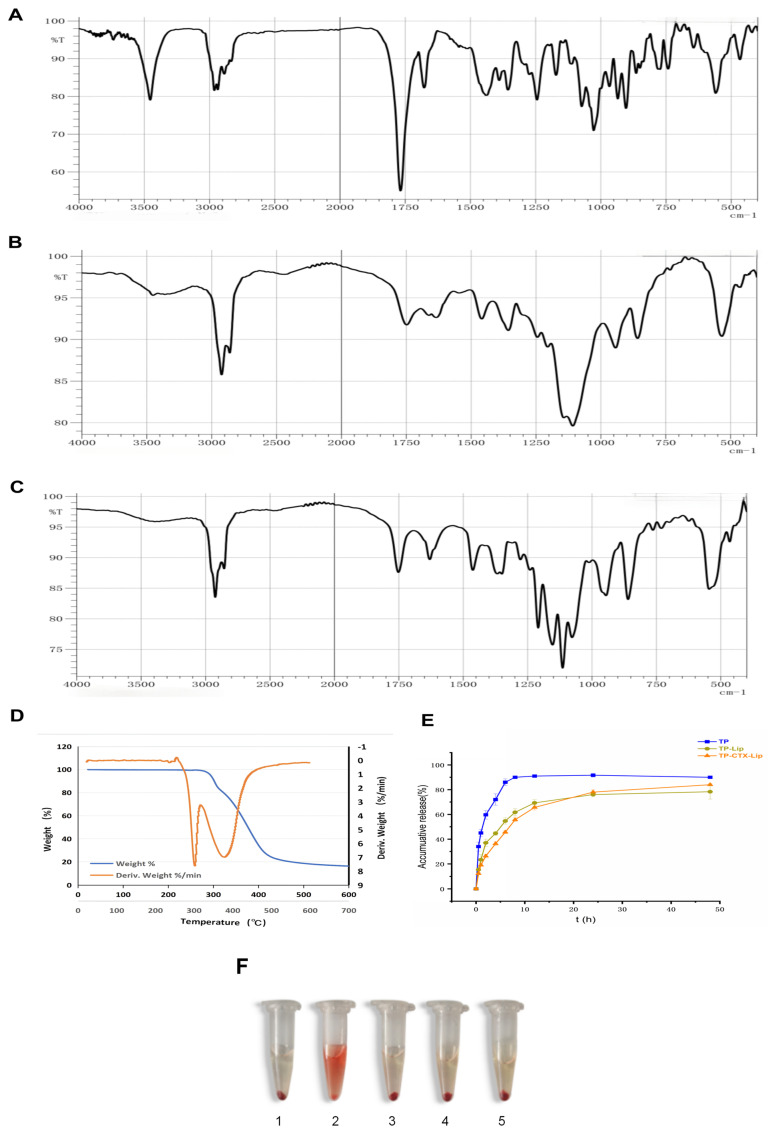
Infrared spectra, thermogravimetric analysis, drug release kinetics, and biocompatibility assessment of the formulation. (**A**) FTIR spectrum of TP; (**B**) FTIR spectrum of blank liposomes (Blank-Lip); (**C**) FTIR spectrum of TP-CTX-Lip. Comparative analysis of the spectra revealed characteristic shifts and band broadening consistent with the formation of non-covalent interactions between TP and the liposomal bilayer; these findings are indicative of successful encapsulation and robust chemical stability of the formulation. (**D**) TGA curve of TP-CTX-Lip, showing its thermal stability under specific conditions; (**E**) In vitro drug release kinetics of TP-CTX-Lip in buffer at pH 7.4 and 37 °C under simulated physiological conditions; (**F**) In vitro hemolysis assay results of TP-CTX-Lip used to assess blood compatibility, with hemolysis percentages compared to the positive control to evaluate safety.

The encapsulation state of TP in liposomes was characterized by Fourier transform infrared spectroscopy (FTIR). The study also explored the interaction between TP and the liposome membrane. Figure 2A shows the characteristic peaks of TP. These include the ester carbonyl (C=O) stretching vibration peak at 1750 cm^−1^; the epoxy group bending vibration peak in the 900–1000 cm^−1^ region; the C-H stretching vibration peak in the 2800–3000 cm^−1^ region; and the -OH stretching vibration peak at 3500 cm^−1^. Compared to the spectrum of TP, the spectrum of TP-CTX-Lip showed significant changes (Figure 2C). The absorption peak in the 900–1000 cm^−1^ region broadened and shifted to lower frequencies, indicating that the epoxy group of TP interacted with the P=O group of phospholipids. The intensity of the C=O peak at 1750 cm^−1^ significantly decreased, suggesting that hydrogen bonding, van der Waals forces, or hydrophobic interactions caused conformational changes. Additionally, the absorption of the C-H vibration peak in the 2900–3000 cm^−1^ region increased, reflecting the overlap of C-H vibrations between TP and the lipid components. The disappearance of the -OH peak at 3500 cm^−1^ indicates that TP forms intermolecular hydrogen bonds with the membrane components.

The thermal stability and decomposition behavior of TP-CTX-Lip were evaluated by thermogravimetric analysis (TGA) and its derivative technique (DTG) (Figure 2D). The TGA curve (black line) shows the change in sample mass with increasing temperature, while the DTG curve (red line) highlights the temperature at which the maximum mass loss occurs, indicating the main decomposition stage of the material. Within the 0–250 °C range, the TGA curve shows minimal mass loss and no obvious DTG peaks. This indicates that water or volatile substances have hardly evaporated and there is no significant thermal degradation. Therefore, TP-CTX-Lip maintained structural integrity under standard storage and handling conditions, meeting the stability requirements of pharmaceutical preparations. In the 250–400 °C range, the TGA curve drops sharply (about 80% mass loss), indicating strong thermal decomposition. The DTG peak at 300 °C corresponds to the temperature of the maximum decomposition rate. Notably, free TP begins to melt and decompose at 226–227 °C, while in TP-CTX-Lip, this process is significantly delayed. This change in thermal performance is mainly attributed to two factors. First, hydrogenated lecithin and cholesterol provide a physical protective effect. Second, intermolecular interactions exist between the drug and lipids, such as van der Waals forces and hydrogen bonds [35,36,37]. These interactions enhance the thermal stability of TP and contribute to an increase in its melting point. Between 300 °C and 400 °C, the DTG curve shows a trend of peak rise followed by a drop, reflecting the continuous degradation process of lipid components after complete decomposition of TP. This stage is related to the rupture of lipid bilayers and breakage of carbon chains, further indicating that the thermal degradation process at higher temperatures is mainly driven by the lipid matrix. The overall mass loss from 250 °C to 400 °C reflects the synergistic effect of drug and lipid degradation, and after 300 °C, the thermal degradation process driven by lipids becomes more significant. This thermal degradation pathway of TP-CTX-Lip indicates that high-temperature conditions may simultaneously induce drug release and lipid decomposition. The results of TGA and differential scanning calorimetry (DSC) confirm that TP-CTX-Lip remains structurally stable below 250 °C, ensuring its suitability for common preparation processes such as filtration and sterilization, as well as long-term storage. Above 300 °C, the decomposition of the lipid backbone reveals the final degradation mechanism, providing valuable guidance for optimizing freeze-drying processes and establishing thermal stability standards.

The encapsulation efficiency (EE) and drug loading (DL) are key parameters for evaluating the performance of liposome formulations. To optimize the preparation conditions of TP-CTX-Lip, a systematic study was conducted on the effects of four influencing factors on encapsulation efficiency and drug loading, while establishing a linear relationship between TP concentration and peak area. The content of TP in liposomes was quantitatively determined by high-performance liquid chromatography (HPLC), thus calculating EE and DL. Within the concentration range of 12.5–500 μg/mL, the peak area of TP showed a strong linear correlation with concentration (Equation (1): R^2^ = 0.9994). This confirms the accuracy, repeatability, and applicability of this method in quantitative analysis.

According to Equation 1, the EE of TP in TP-CTX-Lip is 85.65%. The DL is 13%. These results indicate that a high encapsulation efficiency and optimal drug loading can be achieved when the molar ratio of phospholipids to cholesterol is 6:1.

(2) $${Y\;=\;4\;\times \;10}^{7}x\;+\;175,435$$

The in vitro release curve illustrates the release profiles of TP, TP-Lip, and TP-CTX-Lip in phosphate-buffered solution at pH 7.4 (Figure 2E). TP releases rapidly, with a cumulative release exceeding 90% within 8 h. In contrast, TP-Lip and TP-CTX-Lip exhibit slower, sustained release characteristics, with cumulative release rates of 84.0 ± 4.6% and 79.0 ± 4.0%, respectively, within 24 h. Kinetic analysis indicates that the release profile of TP-CTX-Lip best fits the first-order kinetic model, showing a strong correlation (R^2^ = 0.9676; Table 5). The Higuchi model also fits well (R^2^ = 0.9260). This suggests that diffusion is a major release mechanism, supporting the concept of diffusion-controlled release. In contrast, the zero-order model fits poorly (R^2^ = 0.7628), indicating that zero-order release is unlikely.

The red blood cell (RBC) hemolysis test is a well-established method for evaluating the hemocompatibility of biomaterials. It is generally accepted that a hemolysis rate below 5% indicates good hemocompatibility [38]. In this study, the hemolysis rate of TP-CTX-Lip was quantitatively determined using an ELISA reader to measure absorbance in an ELISA, and the results are presented in Table 6. The hemolysis rate of the negative control group (Tube 1, normal saline) was 0.00%. The hemolysis rate of the positive control group (Tube 2, deionized water) was 100%, confirming the stability and reliability of the experimental system. In the experimental groups, the hemolysis rates of TP-CTX-Lip at concentrations of 25%, 50%, and 75%(*v*/*v*.,Tubes 3 to 5) were 0.85 ± 0.04%, 1.98 ± 0.11%, and 2.73 ± 0.14%, respectively. The hemolysis rates of all experimental groups were significantly lower than the safety threshold of 5%. Figure 2F visually presents the hemolysis conditions of each experimental group, showing that the solution in the negative control group (Tube 1) remained clear, and red blood cells were visible at the bottom of the tube. In contrast, the solution in the positive control group (Tube 2) was uniformly red, indicating complete hemolysis with the release of hemoglobin into the solution. The solutions in the TP-CTX-Lip experimental groups (Tubes 3 to 5) were similar to that of the negative control group, with only slight hemolysis, which was consistent with the quantitative results. This low hemolysis rate may be attributed to the material’s surface properties, such as hydrophilicity and charge neutrality, as well as its components, including the biodegradable carrier [39]. In conclusion, these results indicate that within the tested concentration range, TP-CTX-Lip does not cause significant damage to the red blood cell membrane. It therefore exhibits good hemocompatibility. This provides a crucial foundation for its subsequent in vivo applications, including intravenous injection and other blood-contact treatment methods, and ensures its biological safety.

### 2.2. Evaluation of TP-CTX-LiP Cell Activity

#### 2.2.1. Analysis of Dose and Time Dependency Effects in U87-MG Cells

**Figure 3:**
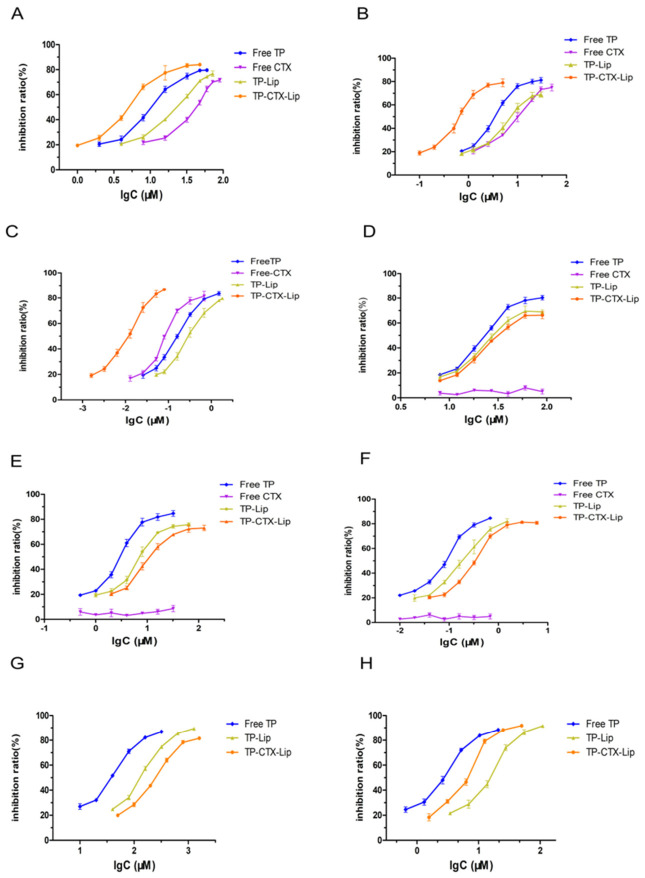

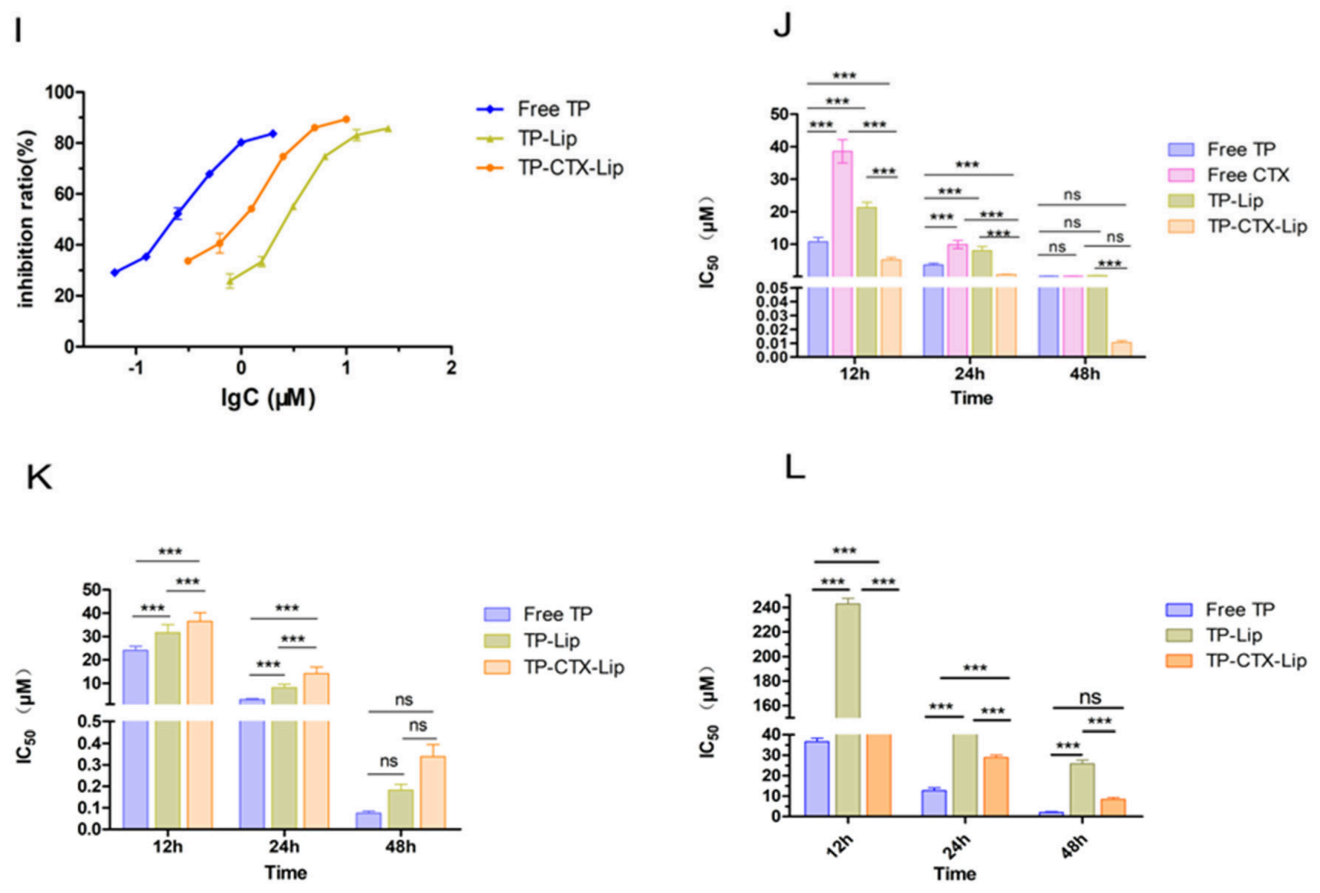
Pharmacodynamic evaluation of TP and its liposomal formulations in glioma cells and non-cancerous cell lines. (**A**–**C**) Dose-dependent inhibitory effects of TP, TP-Lip, and TP-CTX-Lip on SW1088 cells with low epidermal growth factor receptor (EGFR) expression at 12, 24, and 48 h were assessed using the CCK-8 cell viability assay. (**D**–**F**) Dose–response curves for high EGFR-expressing U87-MG cells at 12, 24, and 48 h are shown. These data indicate that the inhibitory effects of the formulations are time-dependent. (**G**–**I**) Dose–response curves for the non-cancerous cell line 3T3-L1 cells at 12, 24, and 48 h are displayed. (**J**) Comparative analysis of the half-maximal inhibitory concentration (IC_50_) of TP and its liposomal formulations at three time points in U87-MG cells revealed that TP-CTX-Lip demonstrated significantly stronger potency at each time point. (**K**) In the comparative analysis of IC_50_ in SW1088 cells, TP-Lip and TP-CTX-Lip showed weaker potency compared to TP. (**L**) TP-Lip and TP-CTX-Lip exhibited lower toxicity in 3T3-L1 cells (statistical significance determined by two-way ANOVA with Tukey’s post hoc test; Mean ± SD, *n* = 3; *** *p* < 0.001, ns, not significant).

In U87-MG cells, the anti-proliferative effects of TP, TP-Lip, and TP-CTX-Lip showed a significant dependence on incubation time (Table 7). For TP, inhibitory activity increased over time, as indicated by the IC_50_ decreasing from 10.71 ± 0.65 μM at 12 h to 0.162 ± 0.008 μM at 48 h. At 12 h, TP-Lip exhibited lower inhibitory activity than TP, with an IC_50_ of 21.27 ± 0.816 μM (*p* < 0.05). TP-CTX-Lip exhibited the strongest inhibitory activity at all time points. At 12 h, its IC_50_ was 5.108 ± 0.380 μM, which is 52.3% lower than that of TP. At 48 h, the IC_50_ further decreased to 0.0104 ± 0.0002 μM. This value is 15.6-fold lower than that of TP and 29.9-fold lower than that of TP-Lip (*p* < 0.001). The dose–response curves (Figure 3A–C) showed concentration-dependent inhibitory effects. Among the treatments, TP-CTX-Lip caused the most significant decrease in cell viability. Notably, the order of inhibitory activity was TP-CTX-Lip > TP > TP-Lip, with significant differences in IC_50_ values among the groups (*p* < 0.05, Figure 3J), demonstrating the enhanced targeting effect mediated by EGFR.

#### 2.2.2. Dose-Dependent and Time-Dependent Effects in SW1088 Cells

In SW1088 cells, the cytotoxicity of TP exhibits a time-dependent increase. The IC_50_ value decreases from 23.940μM at 12 h to 0.075 μM at 48 h, indicating enhanced cytotoxicity over time. TP-Lip exhibits a delayed onset of cytotoxic effects (Table 8). During the early phase (12–24 h), the IC_50_ value of TP-CTX-Lip (36.35 ± 1.878 μM at 12 h) was significantly higher than those of TP-Lip (31.51 ± 1.783 μM) and TP (*p* < 0.05). This observation suggests that the cytotoxic efficacy of TP-CTX-Lip is relatively low at early time points. At the later time point (48 h), the disparity in IC_50_ values between TP-Lip (0.181μM) and TP-CTX-Lip (0.335 μM) diminished, suggesting that despite receptor-related limitations, the encapsulated drug is eventually released (Figure 3D–F). CTX exhibited no detectable toxicity (IC_50_ could not be determined), further confirming the specific cytotoxic activity of TP (Figure 3K). Additionally, the inhibition ratio demonstrates a gradient of cytotoxicity: TP > TP-Lip > TP-CTX-Lip; cytotoxicity decreases with increasing complexity of the formulation.

#### 2.2.3. Dose-Dependent and Time-Dependent Effects in 3T3-L1 Cells

In 3T3-L1 cells, TP exhibited time-dependent cytotoxicity. The IC_50_ values decreased from 10.71 ± 0.650 μM at 12 h to 2.173 ± 0.181 μM at 48 h, confirming the time-dependent nature of its cytotoxicity. After liposomal encapsulation, the cytotoxicity of TP was reduced (Table 9), and the IC_50_ values of both TP-Lip and TP-CTX-Lip were significantly higher than those of TP (*p* < 0.001). This observation indicates that TP-CTX-Lip exhibited reduced cytotoxicity at all time points (Figure 3L).

#### 2.2.4. Investigation of the Correlation Between Drug Efficacy and Treatment Duration as Well as Epidermal Growth Factor Receptor (EGFR) Expression Level

**Figure 4:**
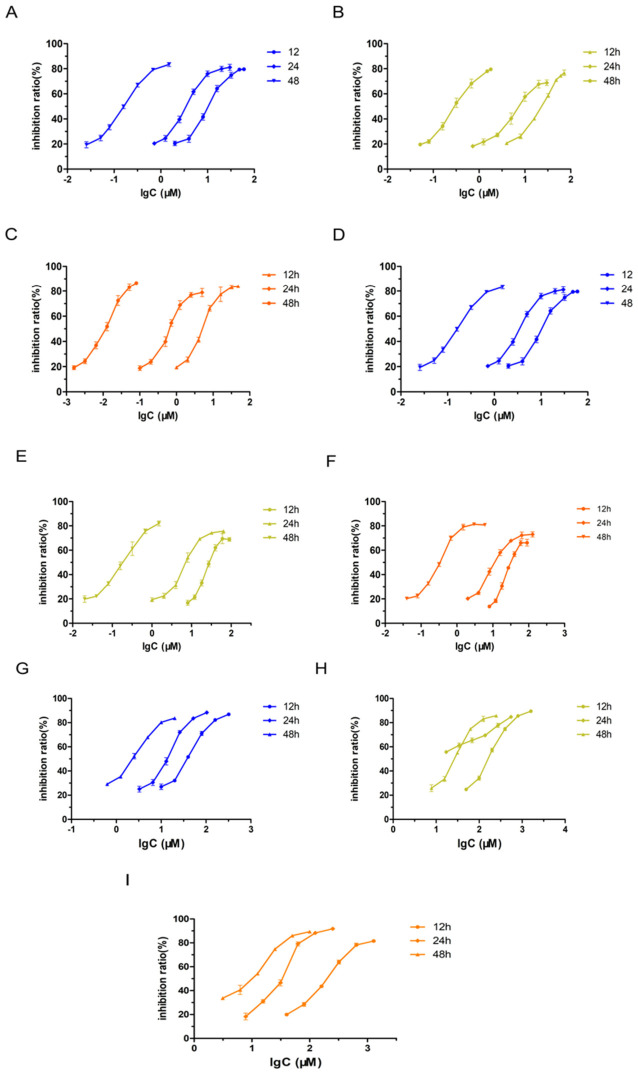
Time-dependent pharmacodynamic analysis of TP and its formulations. (**A**–**C**) Time-dependent inhibitory effects of TP formulations on U87-MG cells (high EGFR expression, exposure duration: 0–48 h): (**A**) Free TP (concentration range: 0.026–60 μM). (**B**) TP-Lip (concentration range: 0.052–72 μM). (**C**) TP-CTX-Lip (concentration range: 0.0016–48 μM). (**D**–**F**) Time-dependent inhibitory effects in SW1088 cells (low EGFR expression, exposure duration: 0–48 h): (**D**) Free TP (0.01–90 μM). (**E**) TP-Lip (0.002–90 μM). (**F**) TP-CTX-Lip (0.004–90 μM). (**G**–**I**) Time-dependent cytotoxicity in 3T3-L1 cells (non-cancerous control; exposure duration: 0–48 h): (**G**) Free TP (2.173–35.63 μM). (**H**) TP-Lip (25.78–242.9 μM). (**I**) TP-CTX-Lip (8.433–126.7 μM). (Mean ± SD, *n* = 3).

Two key factors influencing therapeutic efficacy are treatment duration and Epidermal Growth Factor Receptor (EGFR) dependency [40]. TP and all its formulations showed time-dependent decreases in IC_50_ across three cell lines (Figure 4A–I). However, significant variations were observed in the absolute IC_50_ values among these formulations. For example, the 48 h IC_50_ of TP-CTX-Lip was 0.0104 μM in U87-MG cells and 0.335 μM in SW1088 cells. In the non-cancerous 3T3-L1 cell line, the 12 h, 24 h, and 48 h IC_50_ values of TP-CTX-Lip were 36.35 μM, 28.86 μM, and 8.433 μM, respectively; these values were significantly higher than the corresponding IC_50_ values observed in the TP group.

In addition to treatment duration, therapeutic efficacy also depends on Epidermal Growth Factor Receptor (EGFR) expression. In U87-MG cells, TP-CTX-Lip showed accelerated efficacy in the early stages, with its 12 h IC_50_ significantly lower than that of TP, indicating enhanced EGFR-mediated endocytosis (Figure 4A–C). In SW1088 cells, TP-Lip demonstrated superior efficacy compared to the targeted carrier TP-CTX-Lip (*p* < 0.01, 48 h). This highlights the negative impact of non-specific antibody binding in low-EGFR-expression environments. Notably, TP exhibited greater potency in SW1088 cells (48 h IC_50_: 0.075 μM) compared to U87-MG cells (48 h IC_50_: 0.162 μM) (Figure 4D–F).

#### 2.2.5. U87 Cell Uptake Results

**Figure 5:**
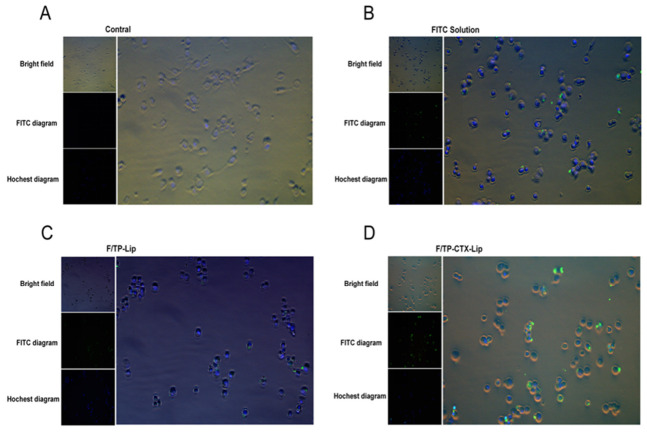

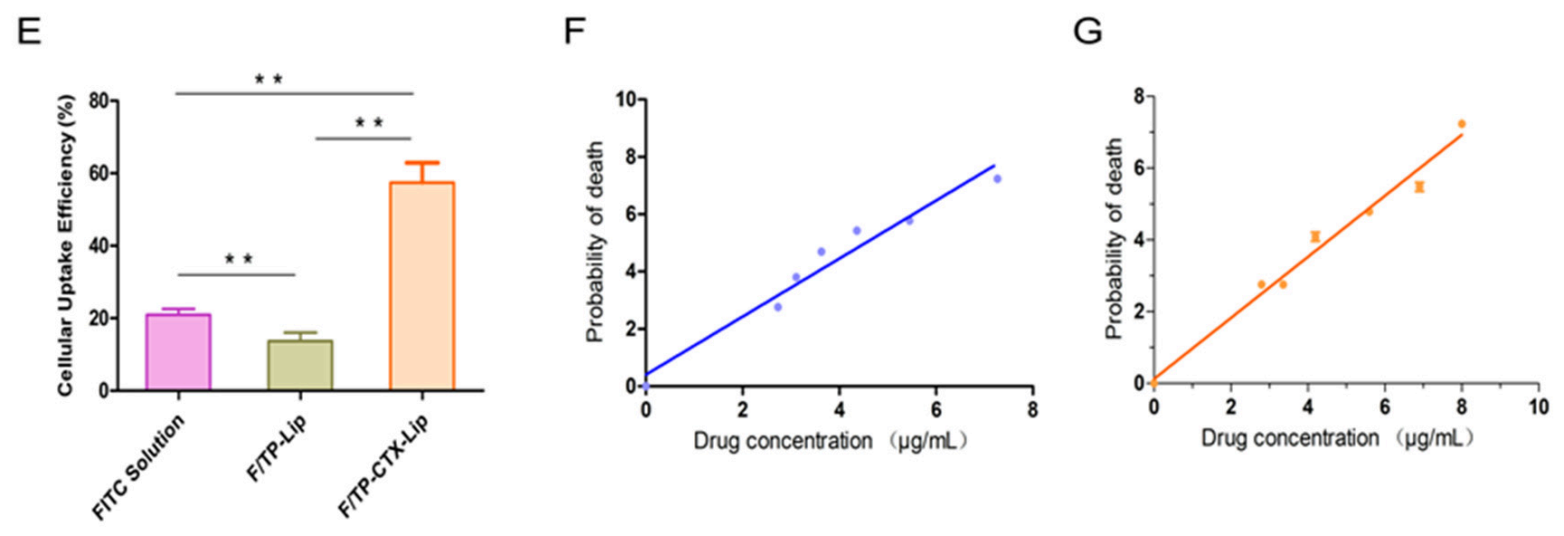
Uptake study of nanoliposomes in U87 cells. (**A**–**D**) Fluorescence images depict the uptake and localization of F/TP-Lip-CTX, F/TP-Lip, and free FITC in U87 cells, Scale bar = 50 μm (×200). (**A**) Fluorescence image showing blank cells; (**B**) Free FITC is distributed throughout the cells; (**C**) F/TP-Lip mainly localized to the cell membrane, with the weakest fluorescence intensity; (**D**) F/TP-Lip-CTX mainly localized to the cytoplasm, with the strongest fluorescence intensity. (**E**) Quantitative analysis of cellular uptake percentage normalized to fluorescence intensity. Quantitative analysis results show significant differences among the three groups (** *p* < 0.01), with the uptake rate of F/TP-Lip-CTX (57.46 ± 5.44%) markedly higher than that of F/TP-Lip (13.7 ± 2.33%) and free FITC (20.97 ± 1.60%). (**F**,**G**) Concentration-dependent developmental toxicity in zebrafish embryos (24 h post-fertilization exposure): (**F**) TP vs. mortality rate. (**G**) TP-CTX-Lip vs. mortality rate (the LC_50_ value of TP-CTX-Lip is 5.733 μg/mL) (Mean ± SD, *n* = 3).

We used FITC as a fluorescent probe to prepare two labeled liposomes, F/TP-Lip and F/TP-Lip-CTX. The uptake behavior of each nanoliposome group in U87 cells was then investigated by detecting the FITC signal. IX71 results showed that after incubation for 30 min, the green fluorescence of F/TP-Lip-CTX within the U87 cells was significantly stronger than that of F/TP-Lip and free FITC solution. F/TP-Lip-CTX was localized mainly in the cytoplasm, exhibiting an uptake rate of approximately 57.46 ± 5.44%. The free FITC solution was distributed throughout the entire cell, with an uptake rate of 20.97 ± 1.60%. In contrast, F/TP-Lip exhibited the lowest uptake, approximately 13.7 ± 2.33%, and was localized mainly on the cell membrane. (Figure 5A–D) The cellular uptake percentage of nanoliposomes was calculated based on data normalized by fluorescence intensity. The three groups showed significant differences (** *p* < 0.01), indicating a marked increase in the uptake rate of F/TP-Lip-CTX (Figure 5E).

#### 2.2.6. Assessment of Embryonic Toxicity in Zebrafish

Zebrafish, as an important model organism in oncology and toxicology research, is widely used due to its transparent embryos, rapid reproductive cycle, highly conserved genome, and similar drug metabolism characteristics to mammals. Studies on the practicality of glioblastoma modeling have shown that injecting approximately 1000 fluorescently labeled U87 cells (RFP+) into zebrafish embryos results in invasive tumor nodules occupying approximately 20% of the brain area and reducing the embryo survival rate to 40% [40,41]. This model provides a reliable platform for evaluating the safety and efficacy of anti-tumor drugs.

Using this zebrafish model, we evaluated the toxicity of both TP and TP-CTX-Lip, which showed concentration-dependent effects in zebrafish embryos. Specifically, the data show that TP at a concentration of 7.27 µg/mL caused 100% mortality (all 20 embryos died). At a concentration of 3.63 µg/mL, the mortality rate dropped to 35% (7 out of 20 embryos died), and no mortality was observed at concentrations ≤2.73 µg/mL (0/20) (Appendix A.1 Table A1). The median LC_50_ of TP was calculated through probit regression analysis as 4.54 µg/mL. This is equivalent to a 516-fold dilution of the stock solution. This low LC_50_ value indicates the strong embryonic toxicity of TP. This value was derived from the probit regression curve (Equation (3)), which showed a goodness-of-fit R^2^ of 0.9556. In this analysis, y represents the probit value and x represents the drug concentration (Figure 5F).

(3) $$\begin{array}{c} Y\;=\;1.012X\;+\;0.4057 \end{array}$$

In contrast, the embryotoxicity of TP-CTX-Lip was significantly reduced (Table 10). At a concentration of 8.0 µg/mL, the mortality rate reached 100% (all deaths), while at 5.6 µg/mL, the mortality rate dropped to approximately 43.3%, and at concentrations ≤4.2 µg/mL, it was relatively lower (about 21.7%). The probability regression analysis method determined that the median LC_50_ of TP-CTX-Lip was 5.733 µg/mL (equivalent to a 37-fold dilution of the original solution concentration) (Equation (4), R^2^ = 0.9780) (Figure 5G). This LC_50_ value was approximately 1.26-fold higher than that of TP, indicating that the liposome encapsulation technology effectively reduced the embryotoxicity of TP. This result established an appropriate safe dose range for subsequent in situ brain tumor transplantation studies in zebrafish.

(4) $$\begin{array}{c} Y\;=\;0.8509X\;+\;0.1221 \end{array}$$

#### 2.2.7. In Vivo Brain Tumor Transplantation and Evaluation of Tumor Suppression Effect

Based on the toxicity assessment results of zebrafish embryos, this study employed an in vivo zebrafish brain tumor transplantation model to further evaluate the inhibitory effects of different formulations on U87-MG tumors. The drug concentrations for each group were determined based on their respective LC_50_ values. The TP group received a TP concentration of 2.27 μg/mL (the original solution diluted 1:1030), which is half of its LC_50_ value. The Blank-Lip group contained no active ingredients. The TP-Lip group received the same concentration of TP as the TP group. The TP-CTX-Lip group was administered 2.87 μg/mL (the original solution diluted 1:74), which corresponds to half of its LC_50_ value, aiming to evaluate the impact of the liposomal delivery system on tumor inhibition.

**Figure 6:**
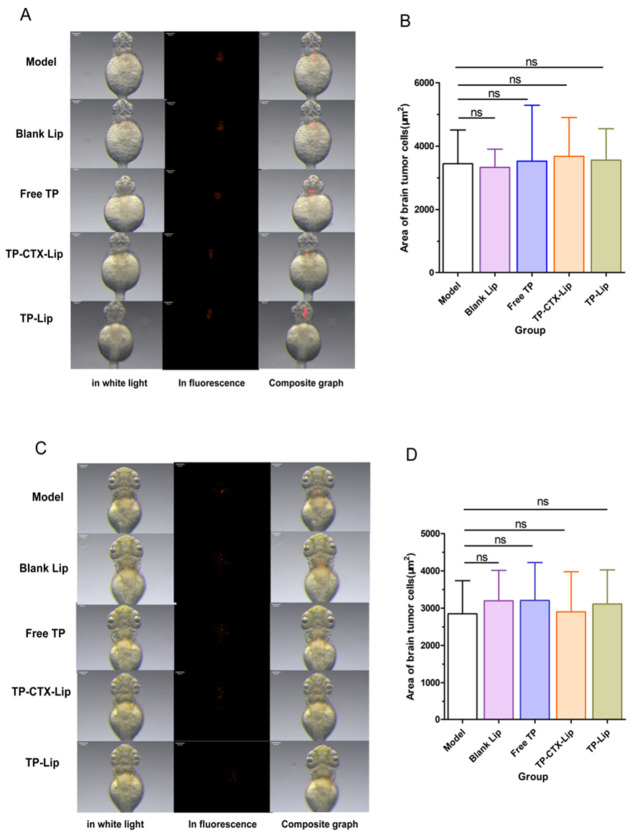

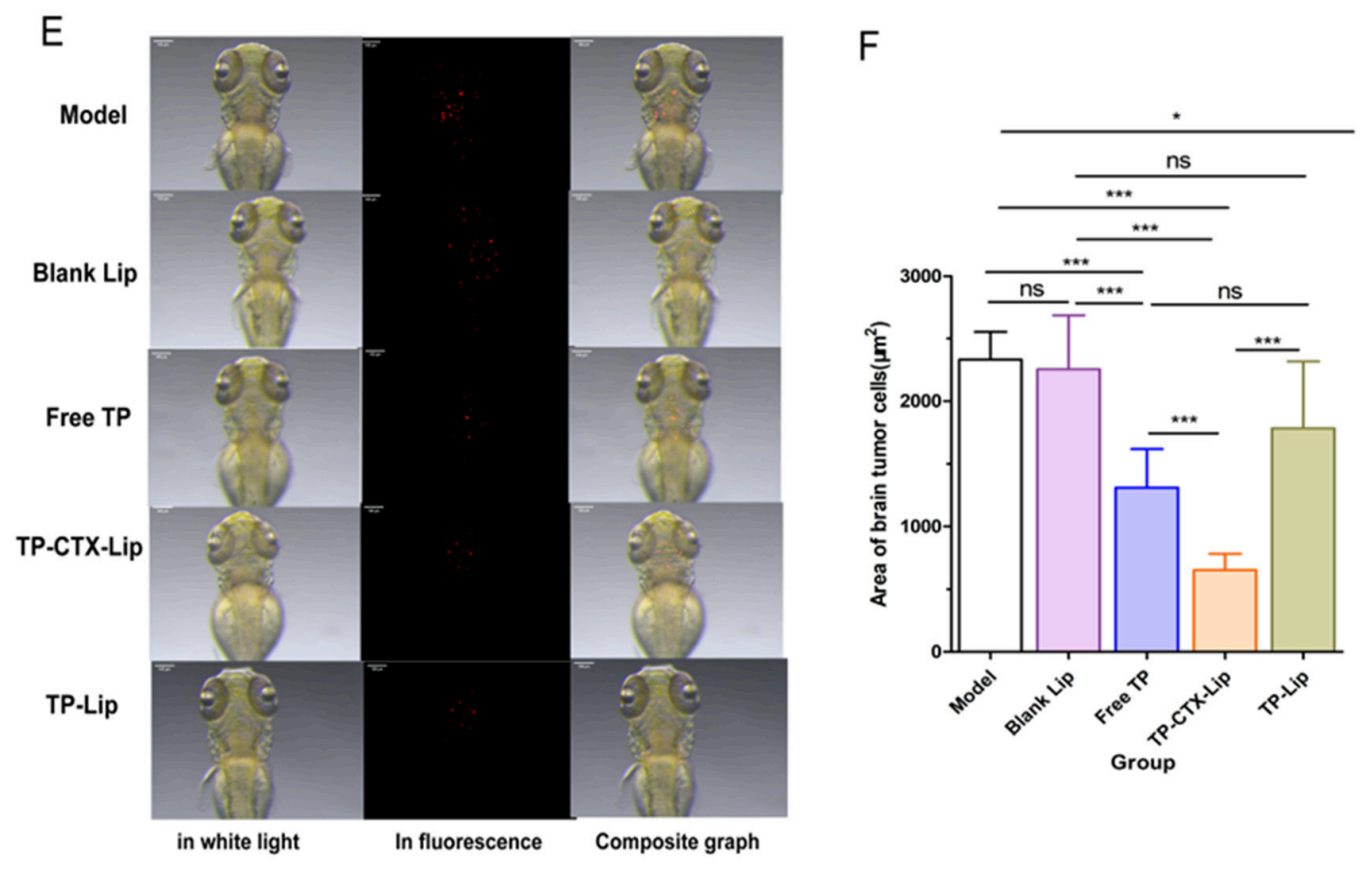
Evaluation of the in vivo anti-tumor activity of TP and its formulations using the zebrafish U87-MG glioma transplantation model with quantitative analysis of tumor area. (**A**) Representative bright-field and dark-field images 2 h post implantation (hpi), showing the initial tumor implantation. (**B**) Statistical results of tumor area at 2 hpi (mean ± SD). (**C**) Representative bright-field and dark-field images at 24 hpi, demonstrating the tumor’s inherent capacity for growth. (**D**) Quantitative analysis of tumor area at 24 hpi (mean ± SD). (**E**) Representative bright-field and dark-field images 24 h after drug treatment, showing a significant reduction in tumor area indicative of a therapeutic response inhibiting tumor growth. (**F**) Statistical analysis of tumor area 24 h after drug treatment (mean ± SD, *n* = 20, * *p* < 0.05, *** *p* < 0.01), ns: non-significant.

U87-MG tumor cells were implanted into the zebrafish brain. At 2 h and 24 h post inoculation, all groups showed a significant increase in tumor area, with no significant differences among them (Appendix A.1 Table A2; Figure 6A,B,D,E), indicating that the tumor transplantation procedure was stable and that the initial inoculation conditions were consistent, thereby laying a reliable foundation for subsequent drug intervention studies.

Drug intervention began 24 h after tumor inoculation, and quantitative analysis of tumor area was conducted for the subsequent 24 h, totaling 48 h from the time of tumor cell inoculation. There was no significant difference between the Blank-Lip group and the normal control group, indicating that the liposomal carrier itself had no obvious inhibitory effect on tumor growth. As a positive control, the tumor area in the TP group was significantly smaller than that in the control group (*p* < 0.0001), demonstrating that TP has strong anti-tumor activity at this concentration. The tumor area in the TP-Lip group was significantly smaller than that in the model group (*p* < 0.005). However, it was still significantly larger than that in the TP group (*p* < 0.0001). This suggests that liposomal encapsulation retained the efficacy of TP. Nevertheless, the drug delivery efficiency or pharmacokinetic properties of this formulation still have room for improvement. Further optimization is required to enhance its performance. Notably, the TP-CTX-Lip group exhibited the most significant anti-tumor effect, with a tumor area significantly smaller than that in the model group (*p* < 0.001) and smaller than that in the TP-only group, showing stronger inhibition (*p* < 0.001). These findings strongly suggest that the addition of CTX may enhance efficacy through a synergistic mechanism (Figure 6C,F; in vivo tumor growth area data can be found in (Appendix A.1 Table A2).

The above results indicate that TP-CTX-Lip shows significant anti-tumor activity in the zebrafish brain in situ tumor transplantation model, superior to the monotherapy group and the liposome-only group. This finding provides experimental evidence for the development of subsequent liposomal combination therapy strategies and also indicates that the zebrafish model has high application value in evaluating the efficacy of brain tumors.

## 3. Discussion

This study successfully developed and evaluated a novel liposome-based combined delivery system (TP-CTX-Lip) for targeted therapy of glioblastoma. The subsequent discussion will elucidate the significance of our key findings, place them within the current scientific context, acknowledge their limitations, and propose future directions for clinical translation.

### 3.1. Interpretation of the Core Findings and Insights at the Mechanism Level

Our main achievement lies in optimizing a stable and high-capacity liposome system, which can effectively address the key drug challenges associated with the potent anti-glioma compound TP. Its sustained release characteristic (in line with first-order kinetics and the Higuchi model) is not only a formulation feature but also the fundamental mechanism for improving therapeutic efficacy. This controlled diffusion through the phospholipid bilayer directly reduces the rapid clearance and low bioavailability issues faced by free TP, thereby transforming its pharmacokinetics from a disadvantage to an advantage [42].

The observed differences in cytotoxicity between SW1088 and U87-MG cells provide strong in vitro evidence for the active targeting ability conferred by CTX. In SW1088 cells, the activity of TP-CTX-Lip was initially inhibited. This inhibition was due to its continuous release profile, which was similar to that of the non-targeted TP-Lip. This similarity confirms that the liposomes themselves were mainly responsible for the altered release kinetics. However, in U87-MG cells, the significantly enhanced efficacy of TP-CTX-Lip at all time points clearly indicates that CTX promotes specific binding and internalization through EGFR-mediated endocytosis [43]. This mechanism is further supported by our cell uptake study, which demonstrated that CTX-conjugated liposomes (F/TP-Lip-CTX) exhibited a significantly higher uptake rate (57.46% ± 5.44%) in U87-MG cells compared to non-targeted liposomes (F/TP-Lip, 13.7% ± 2.33%) or free dye. Notably, fluorescence microscopy revealed that the targeted liposomes were predominantly localized within the cytoplasm, whereas non-targeted liposomes mainly remained associated with the cell membrane. This differential subcellular localization provides preliminary mechanistic evidence that EGFR targeting not only enhances cellular association but also promotes more efficient internalization and intracellular delivery of the payload. This results in more effective delivery of the payload within the cells, overcoming the limitations of passive targeting, which highlights the classic structure–activity relationship of targeted nanomedicines.

### 3.2. Positioning in the Academic Field

Our research findings are consistent with existing nanotherapy models for glioma and have expanded upon them. The use of liposomes to encapsulate chemotherapeutic drugs for more effective delivery to the brain is a widely recognized strategy [44]. Our study further validates the effectiveness of this strategy, demonstrating its superior efficacy in in situ models (the gold standard for assessing brain tumor treatment effects).

However, our research is no longer just a simple encapsulation process; this method incorporates components with active targeting functions. Previous studies have used ligands such as transferrin or peptides, while the use of fully human monoclonal antibody CTX is a more complex approach with high affinity and specificity [45]. There is a significant difference in efficacy between TP-Lip and TP-CTX-Lip in brain tumor models. Moreover, given that the cetuximab-derived targeting ligand used in this study binds to both wild-type EGFR and its constitutively active mutant variant EGFRvIII [46], the developed TP-CTX-Lip strategy may hold potential for treating glioblastoma subtypes characterized by EGFRvIII expression, which is associated with more aggressive disease.

The results of this study emphasize the necessity of active targeting strategies to promote the effective accumulation of therapeutic drugs at the site of brain tumors [47,48]. which stems from the challenge of overcoming the blood–brain barrier, a recognized challenge in the treatment of neuroglioma. We utilized data from the orthotopic transplantation model, which is regarded as the “gold standard” in brain tumor treatment research [49,50]. These data directly demonstrate that CTX-conjugated liposomes have superior anti-tumor efficacy compared to non-targeted liposomes. This finding highlights the key role of active targeting in overcoming the blood–brain barrier. Furthermore, we conducted a systematic evaluation of the toxicity of the nanoparticle delivery system using zebrafish embryo models [51,52]. This model provides a robust, economical, and ethically compliant preliminary screening and toxicity assessment tool during the early stages of development. Our data provide intuitive and quantitative evidence that liposome encapsulation can significantly alter the toxicological characteristics of the loaded drugs, a critical insight often overlooked in early drug development. Notably, the blood–brain barrier of zebrafish embryos is not fully developed. It also has structural and functional differences compared to mammalian blood–brain barriers. Therefore, this study primarily used this model to assess the systemic toxicity and preliminary biodistribution of the nano-preparations. Final conclusions regarding the formulation’s ability to penetrate a mature blood–brain barrier still require validation with data from mammalian models, such as murine orthotopic models. Future research could further explore optimizing the use of and interpretive boundaries of the zebrafish model in neuropharmaceutical delivery studies.

### 3.3. Observed Efficacy and Preliminary Safety Profile of TP-CTX-Lip

Our formulation strategy demonstrated promising results in both efficacy and preliminary toxicity assessments:

Enhanced in situ anti-tumor efficacy: In the orthotopic glioblastoma model, TP-CTX-Lip exhibited superior tumor suppression relative to TP and TP-Lip, indicating its potential for improved therapeutic outcomes.

Reduced cytotoxicity in non-cancerous cells: Consistent with the goal of improving selectivity, in vitro evaluation in the non-cancerous 3T3-L1 cell line showed that liposomal encapsulation, particularly of the targeted TP-CTX-Lip, significantly reduced cytotoxicity compared to free TP across all tested time points. For instance, the 48 h IC_50_ value for TP-CTX-Lip was approximately 4-fold higher than that of free TP, indicating a substantially widened window between effects on cancerous versus non-cancerous cells in this model system.

Favorable preliminary toxicity profile in zebrafish: Assessment in a zebrafish embryo model revealed that TP-CTX-Lip exhibited an increased LC_50_ (by 1.26-fold), a higher LC_50_ value indicating lower toxicity, compared to free TP. Notably, the absolute LC_50_ value (5.733 μg/mL) in zebrafish was substantially greater than the IC_50_ (0.6569 μM) against U87-MG cells in vitro. This substantial separation between the concentrations causing developmental toxicity in zebrafish and those exerting cytotoxic effects on glioma cells in culture is a favorable indication.

Together, these parallel lines of evidence—enhanced efficacy in a relevant disease model, reduced potency in non-cancerous cells, and reduced toxicity in a preliminary vertebrate model—suggest that the TP-CTX-Lip formulation may harbor an improved therapeutic potential compared to the free drug. This supports the concept that formulation engineering can modulate the properties of challenging compounds like TP [53,54,55]. However, a definitive therapeutic index cannot be calculated from these disparate models, and the clinical relevance of these findings requires validation in advanced, pharmacologically relevant mammalian models that integrate both efficacy and toxicity endpoints.

### 3.4. Research Limitations and Future Prospects

Despite the encouraging results of the above studies, some limitations must be acknowledged. First, the differential EGFR expression profiles—specifically, the high expression in U87MG cells and the relatively low expression in SW1088 cells—were adopted from the established literature and were not independently verified in our experimental context using methods such as Western blotting. Secondly, while this study focused on EGFR wild-type models, the targeting ligand employed (cetuximab) is also known to bind to the EGFRvIII mutant. Thus, the current platform could be applicable to EGFRvIII-positive glioblastomas, although this requires direct experimental validation in future studies. Thirdly, the interaction between the TP-CTX-Lip system and a fully normal immune system remains unclear. This interaction is crucial for predicting human responses. Therefore, future research should utilize humanized mouse models. Alternatively, future research could investigate the phenomenon of accelerated blood clearance (ABC). ABC is a process where repeated administration of liposomes leads to their rapid clearance from the bloodstream. Thirdly, although the zebrafish model performs well in preliminary toxicity screening, there are differences between it and mammalian systems in terms of pharmacokinetics and toxicology [55,56]. Following good laboratory practices, comprehensive toxicological studies in rodents are a necessary follow-up step to confirm safety. Finally, while our cell uptake study provides initial insights, the exact intracellular fate of TP-CTX-Lip remains unclear. The specific signaling pathways activated by TP-CTX-Lip in tumor cells remain to be fully elucidated. Future studies should employ fluorescent probes or other tracking methods. Such approaches could enable the real-time observation of the intracellular uptake of liposomes, their escape from the endoplasmic reticulum, and the subsequent drug release.

Considering these findings and limitations, we propose the following future research directions. First, regarding mechanistic studies, proteomic or transcriptomic analyses should be conducted to identify the specific signaling pathways activated by TP-CTX-Lip treatment in tumor cells. Secondly, formulations should be further optimized to explore the incorporation of stimulus-responsive lipids, such as pH-sensitive lipids, thereby achieving drug release triggered in the tumor microenvironment.

## 4. Materials and Methods

### 4.1. The Main Materials and Reagents for the Experiment Are Shown in Appendix A.1, Table A3

All key materials and reagents used in this study are systematically listed in Appendix A.2, Table 1. This table includes information on the chemical names, purities, suppliers, and catalog numbers for each item. The selection of materials was based on established protocols to ensure reproducibility and consistency across experimental replicates.

### 4.2. Cell Lines and Cell Culture

In this study, two human glioma cell lines and one non-cancerous cell line were selected to establish an in vitro model. The first is the U87-MG cell line, which is characterized by high expression of epidermal growth factor receptor (EGFR) [56,57,58]. Compared to the U87-MG cell line, the second selected cell line, SW1088, displays relatively reduced EGFR expression [59]. The third cell line is the mouse embryonic fibroblast 3T3-L1, which serves as a normal control [60]. All cell lines used in this study—the human glioma cell lines U87-MG and SW1088, and the mouse embryonic fibroblast cell line 3T3-L1—were purchased from Zaiji Biotechnology Co., Ltd. (Fuzhou, Fujian, China). All Cells were cultured in high-glucose DMEM supplemented with 10% heat-inactivated fetal bovine serum (FBS) and 1% penicillin-streptomycin. The culture conditions were 37 °C, 5% carbon dioxide, and high humidity. When the cell density reached 70–80%, cells were digested with 0.25% trypsin-EDTA for passaging.

Cell proliferation rate and viability were quantitatively assessed using the CCK-8 kit, following the manufacturer’s instructions. Optical density values were normalized against the untreated control group to calculate the relative viability of the cells.

### 4.3. Collection of Zebrafish Embryos and Establishment of Neuroglial Tumor Models

After collecting AB strain zebrafish embryos through natural mating, the first step was to screen for viable embryos. The screening criteria included three key aspects. First, embryos had to have intact morphology. Second, embryos from the same batch needed to show synchronous development. Third, chorion retraction had to occur at 2 h post-fertilization (hpf). The selected embryos were cultured in E3 medium (5 mM NaCl, 0.17 mM KCl, 0.33 mM CaCl_2_, 0.33 mM MgSO_4_) at 28.5 °C with relative humidity greater than 80%, for up to 72 hpf. On the second day post-fertilization (2 dpf), 0.2 mM 1-phenyl-2-thiourea (PTU) was added to the medium to inhibit melanogenesis. Only embryos exhibiting specific criteria were selected for transplantation. These criteria included a straight body axis, regular heart activity, and an intact yolk sac morphology.

U87-MG cells in the logarithmic growth phase were harvested and processed into a single-cell suspension. The cell suspension was centrifuged at 600× *g* for 5 min at 4 °C. After centrifugation, the supernatant was discarded. The cells were then washed twice. Each wash used 1 mL of calcium- and magnesium-free DPBS. The cells were incubated in serum-free medium in the dark at 37 °C for 8 min and labeled with 5 μM DiI dye. Subsequently, membrane labeling was stabilized by incubation at 4 °C for 15 min. The reaction was terminated by centrifugation at 600× *g* for 5 min at 4 °C, and the labeled cells were suspended in 50–100 μL of cold DPBS at 4 °C for immediate use.

When the embryos reached 2 days post fertilization, they were anesthetized with 0.016% tricaine until gill movement ceased, indicating sufficient anesthesia. We used a Nanoject III micro-injector (Drummond Scientific Company, Broomall, PA, USA) to inject approximately 50–100 cells in a volume of 10 nL. The injection site was located in the forebrain region of the larvae, at coordinates 100 μm posterior to the optic vesicle and a depth of 80 μm. After injection, the larvae were placed individually in a 48-well plate containing E3 medium and cultured in the dark at 31 °C. At 2 h and 24 h post-transplantation (hpt), tumor implantation efficiency was assessed using a Leica M205 FA fluorescence stereomicroscope (Leica Microsystems, Wetzlar, Germany) with excitation at 549 nm and emission at 565 nm. Tumor larvae with a cross-sectional area greater than 2000 μm^2^ were selected for subsequent analysis to ensure experimental consistency.

### 4.4. Preparation of TP-CTX-Lip

In this study, TP-Lip was synthesized using an improved film dispersion method combined with probe ultrasound. First, according to the ratios in Table 1, HSPC and CHOL were dissolved in 5 mL of chloroform, and DSPE-PEG2000-Mal and 0.1% (*w*/*w*) Vitamin E were added. TP was dissolved in methanol at a concentration of 1 mg·mL^−1^. The TP solution was then mixed with the lipid solution. This mixture was sonicated for 5 min at 40 kHz and 300 W. This step formed a uniform organic phase. Subsequently, the organic phase was purged with nitrogen for 10 min to remove residual solvent. After solvent removal, the lipid film was hydrated in pre-warmed PBS (pH 7.4, 60 °C) and fully swollen before undergoing probe ultrasound treatment (600 W, pulse 3 s on/3 s off, ice bath for 2 min). Finally, the solution was filtered 10 times through a 200 nm polycarbonate membrane to obtain monodisperse TP-Lip.

A U_6_ (6^4^) uniform design matrix was used to optimize four key parameters [61] Parameter X_1_ was the concentration of HSPC (0.02–0.07 mmol·L^−1^). X_2_ was the molar ratio of HSPC to CHOL (1:1–6:1). X_3_ was the ratio of HSPC to DSPE-PEG2000-Mal (1:1–10:1). Finally, X_4_ was the mass ratio of TP to lipid (1:1–15:1). Six experimental combinations were generated based on columns 1, 2, 3, and 6 of the U_7_ (7^6^) design table (excluding the last row). The response indicators comprise encapsulation efficiency (EE, %), which is to be maximized; particle size (nm); and polydispersity index (PDI), both of which are to be minimized. To integrate these parameters with disparate units into a comprehensive score (Y), the raw values of each indicator are first normalized into dimensionless sub-scores (Y_1_, Y_2_, Y_3_) ranging from 0 to 1. Specifically, for EE (a “higher-is-better” metric), Y_1_ is calculated as EE/100. Conversely, for particle size and polydispersity index (both “lower-is-better” indicators), the sub-scores are calculated according to the equation Yi = (minimum value)/(actual value). In this context, “minimum value” refers to the lowest observed value for that specific indicator across all experimental runs. Given that the three indicators carry equal weight, the comprehensive score (Y) is computed as the sum of the sub-scores, multiplied by 100:

(5) $${Y\;=\;(Y}_{1}{\;+\;Y}_{2}{\;+\;Y}_{3})\;\times \;100$$

The results were analyzed using IBM SPSS 25.0 software for multiple linear regression analysis. Specifically, factors X_1_ to X_4_ were used as independent variables to construct the regression model for the dependent variable Y, as shown in Equation (6). The overall significance of the model was tested through Analysis of Variance (ANOVA). Additionally, the goodness of fit was evaluated by examining the R^2^ value, which confirmed the model’s effectiveness.

(6) $$\begin{array}{c} {Y\;=\;b}_{0}{\;+\;b}_{1}{x\;+\;b}_{2}x_{1\;}{\;+\;b}_{3}x_{2\;}{\;+\;b}_{4}x_{3}{\;+\;b}_{4}x_{4} \end{array}$$

CTX needs to undergo a thiolation reaction with 2-imino-thiolane (2-IT). In this reaction, 2-IT modifies the thiol group (-SH) on the cysteine residues in the Fc region of CTX. This modification allows CTX to subsequently complex with TP-Lip [61,62]. The reaction was carried out in phosphate-buffered saline (PBS) containing ethylenediaminetetraacetic acid (EDTA) (pH 8.0) at 25 °C in the dark with stirring at 100 rpm for 12 h. After the reaction, the product was dialyzed using a dialysis membrane with a molecular weight cut-off (MWCO) of 1 kDa in PBS containing EDTA (pH 6.0), resulting in the synthesis of CTX-SH. The Ellman method was used to quantitatively determine the thiol content in CTX-SH [63]. The sample reacted with 0.1 mmol L^−1^ 5, 5′-dithiobis- (2-nitrobenzoic acid) (DTNB) in Tris-HCl buffer at pH 8.3. The absorbance was measured at 412 nm and compared with a standard curve of L-cysteine in the concentration range of 0–0.40 mmol L^−1^.

Under a nitrogen atmosphere, the reaction was conducted in the dark at 25 °C for 6 h, where CTX-SH was coupled with maleimide-modified TP-Lip at an initial input molar ratio of DSPE-PEG2000-Mal:CTX-SH = 10:1. Subsequently, the product was purified by size exclusion chromatography (SEC) using a dextran-based gel chromatography column, with PBS as the eluent at a flow rate of 0.5 mL/min. The collected liposome fractions were analyzed for component purity using HPLC, with Blank-Lip as a negative control.

Five milliliters (mL) of the prepared Blank-Lip, TP-Lip, and TP-CTX-Lip were transferred to small vials and freeze-dried at –50 °C under vacuum for 12 h. After the samples were lyophilized to a powder, they were sealed and stored in a dry environment.

### 4.5. Physical and Chemical Properties of TP-CTX-Lip

#### 4.5.1. Particle Size and ζ Potential Analysis

The sizes and ζ potentials of Blank-Lip, TP-Lip, and TP-CTX-Lip were determined at 25 °C by dynamic light scattering (DLS) using a ZetaPlus Z300 analyzer (Particle Size System, Port St. Lucie, FL, USA). Each sample was measured in triplicate. Prior to measurement, 1 mL of each liposome formulation was diluted to 100 mL with phosphate-buffered saline (PBS, pH 7.4) to minimize measurement errors caused by multiple scattering.

#### 4.5.2. Transmission Electron Microscope (TEM) Imaging

The morphology of liposomes was further characterized using a transmission electron microscope (JEM-1400Flash, JEOL, Akishima, Tokyo, Japan). First, 10 μL of lipid suspension (Blank Lip, TP-Lip, or TP-CTX-Lip) was dropped onto a 100-mesh carbon film copper grid, left for 1 min, then excess liquid was absorbed with filter paper and dried. Next, the copper grid was stained with 0.5% (*w*/*v*) phosphotungstic acid (pH 4.5) for 2 min; then, it was naturally dried at room temperature for 1 h. Finally, the transmission electron microscope parameters were set. The accelerating voltage was 80 kV, and the aperture diameter was 20 μm. Images were then collected for each sample. Magnifications ranged from 20,000 to 100,000 times across at least 10 fields of view. This was done to verify particle size distribution and the integrity of the liposome bilayer structure.

#### 4.5.3. DL and EE of TP-CTX-Lip

One milliliter of TP-CTX-Lip suspension was purified using gel permeation chromatography. Liposomes were separated using 5 mL of PBS (pH 7.4) as the eluent. The collected eluent was mixed with 15 mL of methanol, sonicated (300 W; 30 s pulse interval; total duration of 5 min), and then centrifuged (15,000× *g*, 30 min, 4 °C) to obtain the supernatant. The supernatant was concentrated by rotary evaporation; the residue was re-dissolved in methanol and then filtered through a 0.22 μm membrane filter to obtain the encapsulated drug sample.

To determine the total drug content, an equal volume of TP-CTX-Lip suspension was taken, and 4 mL of methanol was added. Under the same sonication conditions, the emulsion was disrupted and then filtered through a 0.22 μm membrane filter.

Quantitative analysis of TP-CTX-Lip was performed using high performance liquid chromatography (HPLC). A calibration curve was established using the external standard method (Y = aX + b, where Y is the peak area, X is the concentration, and a and b are the coefficients of the regression equation).

The method was validated as follows:

Precision: The relative standard deviation (RSD) for intra-day (6 replicates at low, medium, and high concentrations) and inter-day (over 3 consecutive days) measurements was less than 5%;

Accuracy: The recovery rate measured by adding standard substances to blank-lip ranged between 98.2% and 102.4%;

Stability: No significant degradation was observed after storage at 4 °C for 12 h (analyzed at 0, 4, 8, and 12 h).

The calculation methods for DL and EE are as follows:

(7) $$\begin{array}{c} \mathrm{DL}\;(\%)\;=\;(\frac{\mathrm{Mass}\;\mathrm{of}\;\mathrm{encapsulated}\;\mathrm{TP}\;}{\mathrm{Total}\;\mathrm{mass}\;\mathrm{of}\;\mathrm{freeze}-\mathrm{dried}\;\mathrm{nanoparticles}})\;\times \;100\%\; \end{array}$$

(8) $$\begin{array}{c} \mathrm{EE}\;(\%)\;=\;(\frac{\mathrm{Mass}\;\mathrm{of}\;\mathrm{encapsulated}\;\mathrm{TP}\;}{\mathrm{Total}\;\mathrm{mass}\;\mathrm{of}\;\mathrm{initially}\;\mathrm{added}\;\mathrm{TP}})\;\times \;100\%\; \end{array}$$

where encapsulated TP mass = (measured concentration×volume), and total TP mass = (initial input × volume).

#### 4.5.4. FTIR of TP-Lip and TP-CTX-Lip

The chemical interactions between the encapsulated drugs and the lipid carriers were studied using Fourier Transform Infrared Spectroscopy (Nicolet iS50, Thermo Fisher Scientific, Waltham, MA, USA). The freeze-dried formulations TP-Lip and TP-CTX-Lip were analyzed in transmission mode. The analysis parameters were as follows: spectral range 4000–400 cm^−1^; resolution 4 cm^−1^; each sample was scanned 32 times. All spectra were baseline-corrected and corrected for atmospheric interference. Subsequently, a comparative analysis of characteristic functional group vibrations was conducted.

#### 4.5.5. TGA and DTG of TP-CTX-Lip

The thermogravimetric characteristics of the freeze-dried formulations were analyzed using a thermogravimetric analyzer (Q500, TA Instruments, New Castle, DE, USA). Approximately 5 mg of the sample was placed in a platinum crucible and heated according to the following temperature program: from 25 °C to 300 °C at a rate of 10 °C/min, with nitrogen gas as the purge gas at a flow rate of 40 mL/min. Isothermal conditions were maintained at 300 °C for 5 min while recording the mass loss during both dynamic heating and isothermal stages (expressed as a percentage). The resulting data were used to evaluate the dehydration behavior and the stability of excipients and to generate DTG curves for determining key decomposition temperatures.

#### 4.5.6. In Vitro Release Kinetics of TP-CTX-Lip

Using a dialysis membrane with a molecular weight cutoff of 1000 Da, the small-molecule drug TP freely passes through the dialysis membrane into the external medium, while liposomes are retained. By using a sufficient volume of release medium (200 mL), the maximum concentration gradient is continuously maintained to maintain the sink conditions. The in vitro release behavior of TP-CTX-Lip was evaluated in PBS to simulate physiological conditions. The PBS had a pH of 7.4, a concentration of 0.01 M, and contained 0.9% NaCl [64]. Prior to use, the release medium was sonicated for 15 min. This step removed dissolved oxygen and prevented oxidative degradation [4]. Subsequently, the release medium is maintained at a constant temperature of 37 ± 0.5 °C.

To simulate hemodynamic conditions, dialysis bags containing 2 mL of TP-CTX-Lip, TP-Lip, or TP were placed in the release medium. The medium was then stirred magnetically at 100 rpm. At predetermined time intervals (ranging from 0.5 to 48 h), 10 mL samples were collected from the release medium. An equal volume of fresh release medium was added to replenish the system. After filtering the samples through a 0.22 μm polyethersulfone membrane, analysis was performed using HPLC. The calculation method for the cumulative drug release rate is as follows:

(9) $$\begin{array}{c} \mathrm{Cumulative}\;\mathrm{Release}\;\left(\%\right)\;=\;\frac{\sum_{i=1}^{n}\;\left(C_{i}\;\times \;V_{0}+C_{j}\;\times \;V_{j}\right)}{M_{\mathrm{total}}}\times 100\% \end{array}$$

where C_i_ is the TP concentration in the i-th sample (μg/mL), V_0_ is the total medium volume (200 mL), V_j_ and C_j_ represent the volume and concentration of withdrawn samples, respectively, and Mtotal denotes the initial drug load (μg).

#### 4.5.7. Hemolysis Test to Evaluate the Blood Compatibility of TP-CTX-Lip

The hemolysis rate of TP-CTX-Lip was assessed according to standard procedures [33]. A total of 0.5 mL of heparinized mouse whole blood was centrifuged at 2000× *g* for 5 min at 4 °C to separate the red blood cell pellet. The pellet was washed three times with 1 mL of 0.9% sodium chloride solution and centrifuged after each wash to remove plasma impurities. A 2% red blood cell suspension was prepared by mixing 100 μL of the red blood cell pellet with 490 μL of 0.9% sodium chloride solution.

Prepare each experimental group by mixing 500 μL of 2% red blood cell suspension with the volumes of TP-CTX-Lip and 0.9% NaCl solution specified in Table 11. The specific concentrations and volume ratios used in each experimental group are detailed in Table 11; all experiments were performed in triplicate (*n* = 3). All samples were incubated at room temperature for 2 h and then centrifuged at 3000× *g* for 5 min at 4 °C. Then, 100 μL of the supernatant was collected to measure absorbance at 540 nm. The hemolysis rate (%) was calculated using Formula (10) as shown:

(10) $$\begin{array}{c} \mathrm{Hemolysis}\;(\%)\;=\;\frac{{\mathrm{OD}}_{\mathrm{Sample}}-{\mathrm{OD}}_{\mathrm{Negative}\;}}{{\mathrm{OD}}_{\mathrm{Positive}}-{\mathrm{OD}}_{\mathrm{Negative}}}\;\times \;100\% \end{array}$$

In this formula, OD_sample_ denotes the absorbance of the test samples. OD_negative_ denotes the absorbance of the negative control (0.9% NaCl). OD_positive_ denotes the absorbance of the positive control (deionized water).

### 4.6. TP-CTX-Lip Cell Toxicity Assay

U87-MG cells were seeded in a 96-well plate at a density of 9 × 10^3^ cells per well and cultured for 24 h. The experiment began when the cell confluence, estimated by microscopic observation, exceeded 90% and the cells were in the logarithmic growth phase. Before drug treatment, the cells were washed twice with precooled PBS to avoid serum interference. The experiment was divided into treatment and control groups. The treatment groups included TP/TP-Lip (4.583–138.889 μM, six logarithmically spaced concentrations) and TP-CTX-Lip (0.046–4.167μM, seven logarithmically spaced concentrations). The control group consisted of solvent or complete culture medium alone. All experimental conditions were performed in triplicate.

Cytotoxicity assessment: After 48 h of drug treatment, the culture medium was replaced. The new medium contained 10% (*v*/*v*) CCK-8 reagent. Cells were then, incubated with this reagent for 2 h, protected from light. Following incubation, the absorbance at 450 nm was measured using a BioTek Synergy H1 microplate reader (BioTek Instruments, Winooski, VT, USA). Cell viability was calculated as described in Equation (11).

(11) $$\begin{array}{c} \mathrm{Cell}\;\mathrm{Viability}\;\left(\%\right)\;=\;\left(\frac{{\mathrm{OD}}_{\mathrm{treatment}}-{\mathrm{OD}}_{\mathrm{blank}}}{{\mathrm{OD}}_{\mathrm{control}}-{\mathrm{OD}}_{\mathrm{blank}}}\right)\;\times \;100\% \end{array}$$

where OD_treatment_, OD_control_, and OD_blank_ represent the absorbance of drug-treated wells, vehicle control wells, and blank (medium-only) wells, respectively.

### 4.7. Cell Uptake Study

FITC served as a fluorescent probe to prepare FITC-labeled F/TP-Lip and F/TP-Lip-CTX, with free FITC solution used as a control. U87-MG cells were seeded into 12-well plates at a density of 2 × 10^5^ cells per well and cultured for 24 h at 37 °C under 5% CO_2_. After removing the culture medium, fresh medium containing either diluted FITC solution or FITC-labeled TP nanoliposomes was added; the final FITC concentration was kept at 20 ng/mL in all groups. Following a 0.5 h incubation, the medium was aspirated, and cells were washed three times with PBS (pH 7.4). Nuclei were then stained with Hoechst for 15 min. Cellular uptake was visualized using an inverted phase-contrast fluorescence microscope (IX71; Olympus Corporation, Shinjuku, Tokyo, Japan).

### 4.8. Experimental Studies in Zebrafish

The AB strain of zebrafish used in this experiment was obtained from the National Zebrafish Resource Center in China. All zebrafish experimental protocols were conducted in accordance with the ethical guidelines established by the Institutional Animal Care and Use Committee (IACUC) of China Fuzhou Cold-Spring Biology Co., Ltd, Fzhou, China). The IACUC issue number assigned was IACUCBWS-2025011506, and the approval number was IACUCBWS-PZ202527. Embryos were obtained by natural mating. Detailed information on the reagents and solutions used for zebrafish cultivation can be found in Appendix A.1, Table A3, and Appendix A.2.

#### 4.8.1. The Reagents for Zebrafish Culture and Zebrafish Embryo Preparation

Healthy zebrafish embryos were selected based on the following three criteria: (1) normal embryonic morphology; (2) synchronization in developmental stage; and (3) observation of contraction of the embryo membrane within 2 h post fertilization. Contraction of the embryo membrane is an important physiological indicator of embryo health [65]. After selection, the embryos were cultured in E3 culture medium. The composition was as follows: 5 mM sodium chloride, 0.17 mM potassium chloride, 0.33 mM calcium chloride, and 0.33 mM magnesium sulfate. Culture conditions were maintained at 28.5 °C with relative humidity above 80%. This culture lasted for 72 h post fertilization.

#### 4.8.2. Dose-Dependent Toxicity Assessment

Embryos were treated with 0.003% (*w*/*v*) sodium hypochlorite solution for the first 72 h of development. After treatment, the embryos were placed in a 6-well plate, with 20 embryos in each well. Then, 3 milliliters of E3 medium were added to each well. For each treatment group, six different concentrations were prepared. Each concentration had three biological replicates. The drugs were administered by immersion. The groups included the blank control group (E3 medium only), the TP group (0.25–0.67 μg/mL), the TP-Lip group (0.25–0.67 μg/mL), and the TP-CTX-Lip group (2.22–13.33 μg/mL). The embryos were cultured in a dark environment at 28.5 °C. Cardiac arrest was used as the criterion for assessing mortality; the mortality rate was recorded, and deceased embryos were promptly removed. The experiment lasted 24 h, after which the cumulative mortality rate was calculated. A survival rate of ≥90% in the blank control group was required to validate the experiment. The maximum tolerated dose (MTD) was determined by plotting the dose–response curve.

#### 4.8.3. Inhibit the Proliferation Activity of Tumor Cells in Zebrafish Bodies

Collect U87-MG cells in the logarithmic growth phase, prepare them into a single-cell suspension, and centrifuge at 600× *g* for 5 min at 4 °C. After discarding the supernatant, gently vortex the cells twice with 1 mL of DPBS without calcium and magnesium ions each time. Incubate the cells in serum-free medium at 37 °C for staining in the dark for 8 min, then perform cell membrane stabilization treatment at 4 °C for 15 min. Stop the staining reaction by centrifugation at 600× *g* for 5 min at 4 °C, and resuspend the labeled cells in 50–100 μL of cold DPBS for transplantation.

Embryo preparation and selection: Embryos of the AB strain at 2 days post fertilization were cultured in E3 medium supplemented with 0.2 millimolar paraformaldehyde to inhibit pigmentation. Selection criteria included (1) a straight body axis; (2) regular heartbeat; (3) complete yolk sac morphology.

Cell transplantation: Embryos were anesthetized with 0.016% tetracaine until gill movement ceased. Inject 10 nanoliters of DiI-labeled U87-MG suspension into the anterior brain region (100 μm posterior to the eye sac and 80 μm deep) using a Nanoject III micropipette (Drummond Scientific Company, Broomall, PA, USA).

Post-transplantation analysis: The injected larvae were placed in 48-well plates. They were then cultured in a 31 °C dark environment containing E3 medium. The success rate of transplantation was evaluated by microscopy at 2 and 24 h post injection. The microscope used was a Leica M205 FA, (Leica Microsystems, Wetzlar, Germany) with excitation at 549 nm and emission at 565 nm. Larvae with tumor areas larger than 2000 μm^2^ were selected for further study. This selection ensured uniformity in tumor formation.

The zebrafish used to establish brain xenografts were randomly assigned to experimental groups using a computer-generated randomization system (Table 9). A total of 240 zebrafish were used and divided into four groups of 60 fish each. Each group comprised three biological replicates of 20 zebrafish. All groups were cultured at 32 °C with humidity controlled at 70% ± 5%, using E3 medium supplemented with 0.003% methylene blue (an antibiotic). The medium was changed daily.

Administration protocol: The drug concentration was standardized at half the LC_50_ value derived from the toxicity curve of the previous experiment:

(1) Model control group: E3 medium containing 0.1% dimethyl sulfoxide;
(2) TP group: TP solution at half the LC_50_ dose;
(3) TP-Lip group: Liposomes containing the same concentration of TP as the TP group;
(4) TP-CTX-Lip group: CTX-modified immunoliposomes.

The TP concentration in all preparations was verified using HPLC. The treatment duration was 48 h.

Fluorescence imaging: At the end of the experiment, 10 zebrafish from each group were randomly selected for visualization of tumors. The brain graft tissue was imaged at 8× magnification using a stereoscopic fluorescence microscope (Leica M205 FA), (Leica Microsystems, Wetzlar, Germany) with consistent exposure settings for all samples.

Image processing: The tumor area was quantified using ImageJ software (Rasband, W.S. ImageJ, version 1.8.0; ImageJ.js variant used for data analysis in this study; National Institutes of Health: Bethesda, MD, USA, 2024). with a pre-calibrated 8× magnification factor.

## 5. Conclusions

In summary, this study successfully developed and optimized an EGFR-targeted liposomal formulation, TP-CTX-Lip, for the delivery of triptolide. The system combines the sustained release profile of a nanocarrier with the specificity of cetuximab-mediated targeting. In vitro evaluations demonstrated that TP-CTX-Lip exhibited potent and selective cytotoxicity against high-EGFR U87-MG glioma cells, while showing significantly reduced toxicity in non-cancerous 3T3-L1 cells. Enhanced cellular uptake mediated by EGFR targeting was confirmed as a key mechanism contributing to its efficacy. In a zebrafish orthotopic glioblastoma model, TP-CTX-Lip effectively suppressed tumor growth and concurrently reduced systemic developmental toxicity, collectively resulting in a markedly improved therapeutic window. These findings provide a solid preclinical foundation for the development of TP-based targeted nanomedicines and underscore the potential of formulation engineering to overcome the clinical translation barriers of potent but challenging natural compounds like triptolide.

## Figures and Tables

**Table 1:** Uniform design experimental layout and comprehensive scores.

Experiment	X_1_ (mmol)	X_2_ (HSPC:CHOL M:M)	X_3_ (HSPC:DSPE-PEG2000-Mal M:M)	X_4_ (HSPC:TP m:m)	Comprehensive Score (Y)
1	0.04	5	2.0	15	254.0
2	0.05	3	0.0	10	238.0
3	0.06	1	1.5	8	262.5
4	0.07	6	5.0	4	286.0
5	0.02	4	1.0	2	227.5
6	0.03	2	2.5	1	229.0

**Table 2:** Model coefficients for comprehensive score regression.

Model	Unstandardized Coefficient		Standardized Coefficient	t	Significance
	B	Standard Error	Beta		
Constant	190.697	1.269		150.324	0.004
HSPC dosage X_1_	1192.347	24.837	0.986	48.007	0.013
HSPC/CHOL X_2_	3.974	0.227	0.329	17.501	0.036
HSPC/DSPE-PEG2000-Mal X_3_	−2.432	0.135	−0.366	−18.044	0.035
HSPC/TP X_4_	0.024	0.082	0.006	0.292	0.819

Dependent variable: comprehensive score.

**Table 3:** Characteristics of TP-Lip prepared by optimal scheme (*n* = 3).

Indicators	Predicted Value	Measured Value	Deviation Rate
Packaging rate	89.12%	85.83% ± 1.81%	−3.69%
Particle size	125.4 nm	131.2 ± 3.76 nm	+4.63%
PDI	0.212	0.223 ± 0.06	+5.19%
Comprehensive scoring	295	287	−2.71%

**Table 4:** Particle size, zeta potential, and PDI of Blank-Lip, TP-Lip and TP-CTX-Lip.

Name	Size (nm)	Zeta (mV)	PDI
Blank-Lip	103.40 ± 3.90	−33.49 ± 0.22	0.146 ± 0.013
TP-Lip	115.30 ± 3.76	−28.37 ± 1.11	0.223 ± 0.021
TP-CTX-Lip	131.30 ± 4.50	−23.37 ± 0.27	0.240 ± 0.006

**Table 5:** In vitro release model fitting for TP-CTX-Lip.

Release Model	Fitting Curve	Adjusted R^2^	Goodness of Fit
Zero-order	y = 20.89 + 1.47x	0.7628	Not significant
First-order	$y\;=\;80.98{\;\times \;(1\;\times \;10}^{-0.15x})$	0.9676	High degree of fit
Higuchi	$y=12{.08\;\times \;X}^{1/2}\;+\;7.88$	0.9260	fit

**Table 6:** Hemolytic Activity of TP-CTX-Lip at Different Concentrations. (*n* = 3).

Sample	Negative Control	Positive Control	25%TP-CTX-Lip	50%TP-CTX-Lip	75%TP-CTX-Lip
Hemolysis rate (%)	0.00	100.00	0.85 ± 0.04	1.98 ± 0.11	2.73 ± 0.14

**Table 7:** IC_50_ values of tested formulations in U87-MG cells with 95% confidence intervals (*n* = 3).

	12 h		24 h		48 h	
Formulation	IC_50_ (μM)	95% Confidence Interval	IC_50_ (μM)	95% Confidence Interval	IC_50_ (μM)	95% Confidence Interval
Free TP	10.71 ± 0.650	9.511 to 12.060	3.606 ± 0.248	3.151 to 4.125	0.162 ± 0.008	0.147 to 0.179
Free CTX	38.46 ± 1.842	35.01 to 42.23	9.823 ± 0.638	8.650 to 11.150	0.089 ± 0.006	0.077 to 0.102
TP-Lip	21.27 ± 0.816	19.73 to 22.93	7.945 ± 0.652	6.770 to 9.324	0.311 ± 0.018	0.278 to 0.348
TP-CTX-Lip	5.108 ± 0.380	4.417 to 5.908	0.6569 ± 0.053	0.562 to 0.768	0.0104 ± 0.0002	0.009 to 0.001

**Table 8:** Testing the IC_50_ value and 95% confidence interval of the formulation in SW1088 cells (*n* = 3).

	12 h		24 h		48 h	
Formulation	IC_50_ (μM)	95% Confidence Interval	IC_50_ (μM)	95% Confidence Interval	IC_50_ (μM)	95% Confidence Interval
Free TP	23.940 ± 0.989	22.150 to 25.870	2.965 ± 0.229	2.550 to 3.448	0.075 ± 0.005	0.066 to 0.086
Free CTX	---	---	---	---	---	---
TP-Lip	31.510 ± 1.783	28.210 to 35.20	8.051 ± 0.741	6.730 to 9.633	0.181 ± 0.014	0.156 to 0.210
TP-CTX-Lip	36.350 ± 1.878	32.850 to 40.210	13.96 ± 1.385	11.50 to 16.93	0.335 ± 0.029	0.284 to 0.396

**Table 9:** Testing the IC_50_ value and 95% confidence interval of the formulation in 3T3-L1 cells (*n* = 3).

	12 h		24 h		48 h	
Formulation	IC_50_ (μM)	95% Confidence Interval	IC_50_ (μM)	95% Confidence Interval	IC_50_ (μM)	95% Confidence Interval
Free TP	35.63 ± 3.194	32.18 to 39.46	12.350 ± 1.272	10.99 to 13.87	2.173 ± 0.181	1.978 to 2.388
TP-Lip	242.9 ± 18.734	222.6 to 265.0	67.79 ± 7.233	60.10 to 76.47	25.780 ± 2.691	22.920 ± 29.010
TP-CTX-Lip	126.7 ± 9.146	116.8 to 137.5	28.860 ± 2.788	25.880 to 32.190	8.433 ± 0.954	7.422 to 9.582

**Table 10:** Mortality Rate of Zebrafish Embryos in TP-CTX-Lip-Exposed Group (*n* = 20).

Drug Concentration (μg/mL)	Group 1 (Deaths/Total)	Group 2 (Deaths/Total)	Group 3 (Deaths/Total)	Average Mortality Rate (%)	Probability Average Value Y
0	0/20	0/20	0/20	0	0
2.8	0/20	0/20	0/20	0	7.24
4.2	3/20	5/20	3/20	21.67	5.48
5.6	8/20	9/20	8/20	43.33	4.79
6.9	15/20	12/20	14/20	65	4.08
8.0	20/20	20/20	20/20	100	2.76

Note: Data are presented as the number of deaths per group divided by the total number of embryos. Groups 1, 2, and 3 represent three independent parallel replicates at each concentration.

**Table 11:** Grouping design for hemolysis assay.

Test Group	RBC Suspension (μL)	0.9% NaCL (μL)	Deionized Water (μL)	TP-CTX-Lip (μL)
negative	500	500	0	0
positive	500	0	500	0
5% TP-CTX-Lip	500	475	0	25
10% TP-CTX-Lip	500	450	0	50
15% TP-CTX-Lip	500	425	0	75

## Data Availability

The original contributions presented in this study are included in the article. Further inquiries can be directed to the corresponding authors.

## Appendix A

### Appendix A.1

**Table A1:** Mortality Rate of Zebrafish Embryos in TP-Exposed Group (*n* = 20).

Drug Concentration (μg/mL)	Group 1 (Deaths/Total)	Group 2 (Deaths/Total)	Group 3 (Deaths/Total)	Average Mortality Rate (%)	Probability Average Value Y
0	0/20	0/20	0/20	0	0
7.27	20/20	20/20	20/20	100	7.24
5.45	16/20	15/20	16/20	78.33	5.78
4.36	13/20	14/20	13/20	66.67	5.43
3.63	9/20	7/20	7/20	38.33	4.7
3.11	2/20	3/20	2/20	11.67	3.8
2.73	0/20	0/20	0/20	0	2.76

**Note**: Data are presented as the number of deaths per group divided by the total number of embryos. Groups 1, 2, and 3 represent three independent parallel replicates at each concentration.

**Table A2:** Data on Tumor Growth Areas in Zebrafish (μm^2^).

	2 h (Post-Tumor Transplantation)	24 h (Post-Tumor Transplantation)	24 h (After Drug Intervention)
Model	3447.11 ± 1063.98	2846.72 ± 889.28	2331.20 ± 222.32
Blank Lip	3330.66 ± 576.93	3197.72 ± 815.70	2256.56 ± 429.10
Free TP	3527.94 ± 1761.94	3205.55 ± 1018.05	1311.06 ± 306.23
TP-CTX-Lip	3682.19 ± 1219.67	2900.61 ± 1076.94	652.0917 ± 131.25
TP-Lip	3561.61 ± 988.89	3111.27 ± 912.55	1784.94 ± 532.02

**Table A3:** Key resources.

REAGENT or RESOURCE	SOURCE	IDENTIFIER
Triptolide	Shanghai McLyn Biochemical Technology Co., Ltd. Shanghai, China	CAS:38748-32-2 RRID:T819208
Hydrogenated Soybean Phospholipids	Ron’s reagent, Shanghai, China	CAS:92128-87-5 RRID:R095239
Cholesterol	Damas-beta, Shanghai, China	CAS:57-88-5 RRID:P2383457
Cetuximab	Meilunbio, Shanghai, China	CAS:205923-58-4 RRID:D1212C
DSPE-PEG-Male.M.W.2000	Tanshtech, Guangzhou, China	CAS:N/A RRID:80031306-2000
Vitamin E	Xingsha Pharmaceutical Co., Ltd., Xiamen, China	CAS:7695-91-2 RRID:S13017
1× PBS	Solarbio, Beijing, China	Cat:P1020 RRID:240005008
2-Iminothiolane	Adamas-beta, Shanghai, China	CAS:4781-83-3 RRID:59242A
L-Cysteine	Solarbio, Beijing, China	CAS:52-90-4 RRID:1214Z021
Tris-Hcl	Adamas-beta, Shanghai, China	CAS:1185-53-1 RRID:P2290031
DTNB	Adamas-beta, Shanghai, China	CAS:69-78-3 RRID:P2300112
Methanol	Sigma, Shanghai, China	CAS:67-56-1 RRID:I1282407 312
Acetonitrile	Sigma, Shanghai, China	CAS:75-05-8 RRID:JB127930
Cell culture		
U87-MG	KERONG Biotechnology	RRID:KR-RXBZ-1071
SW1088	BNCC Biotechnology	RRID:BNCC341219-1
Characterized Fetal Bovine Serum	Solarbio, Beijing, China	RRID:S9000
DMEM Medium	Pricella, Wuhan, China	RRID:WHB825Z281
Trypsin-EDTA	Solarbio, Beijing, China	RRID:T1300
Cell Counting Kit-8	Solarbio, Beijing, China	RRID:CA1210-500T
Gibco Penicillin-Streptomycin, Liquid	ThermoFisher, Shanghai, China	RRID:15140122
CELLSAVING	Macklin, Shanghai, China	RRID:C854497-50mL
DMSO (Dimethyl sulfoxide)	Solarbio, Beijing, China	CAS:67-68-5 RRID:D8371-50
Zebrafish culture		
NaCl	National Pharmaceutical Group, Beijing, China	CAS:7647-14-5
KCl	National Pharmaceutical Group, Beijing, China	CAS:7447-40-7
Ca (NO_3_)_2_·4H_2_O	National Pharmaceutical Group, Beijing, China	CAS:10035-04-8
CaCl2·2H2O	National Pharmaceutical Group, Beijing, China	CAS:10035-04-8
MgSO4·7H2O	National Pharmaceutical Group, Beijing, China	CAS:10034-99-8
NaOH	National Pharmaceutical Group, Beijing, China	CAS:1310-73-2
NaClO	National Pharmaceutical Group, Shanghai, China	CAS:7681-52-9
HEPES	Aladdin, Shanghai, China	CAS:7365-45-9
MS-222	Sigma, Shanghai, China	CAS 886-86-2
Tricaine methanesulfonate	Sigma, Shanghai, China	CAS:886-86-2
Vybrant™ CM-DiI	ThermoFisher, Shanghai, China	CAS:V22888

### Appendix A.2

Reagents and Solutions for Zebrafish Cultivation

Embryo culture medium: Danieau’s buffer is prepared by dissolving analytical-grade reagents in ultrapure water to a final volume of 200 milliliters. The components include sodium chloride (20.34 g), potassium chloride (0.31 g), calcium sulfate·4H_2_O (0.85 g), magnesium sulfate·7H_2_O (0.59 g), and HEPES (7.12 g). The pH is adjusted to 7.2 ± 0.1 using 1 M sodium hydroxide or hydrochloric acid. The solution is then sterilized by filtration through a 0.22 μm membrane filter. Danieau’s buffer is stored at 4 °C and diluted 100-fold with ultrapure water before use.

Anesthetic: To prepare a 0.03% (*w*/*v*) anesthetic solution, 550–650 μL of MS-222 stock solution (tricaine methanesulfonate) is added to 50 mL of embryo culture medium. Additionally, for instrument sterilization, 5 μL of MS-222 stock solution is mixed with 250 mL of ultrapure water.

Cell labeling: Vybrant™ CM-DiI is used to label U87-MG glioma cells for in vivo tracking.
